# Precision medicine for asthma treatment: Unlocking the potential of the epigenome and microbiome

**DOI:** 10.1016/j.jaci.2024.06.010

**Published:** 2024-06-19

**Authors:** Javier Perez-Garcia, Andres Cardenas, Fabian Lorenzo-Diaz, Maria Pino-Yanes

**Affiliations:** a Genomics and Health Group, Department of Biochemistry, Microbiology, Cell Biology, and Genetics, Universidad de La Laguna (ULL), La Laguna, Tenerife; b Instituto Universitario de Enfermedades Tropicales y Salud Pública de Canarias (IUETSPC), Universidad de La Laguna (ULL), La Laguna, Tenerife; c Instituto de Tecnologías Biomédicas (ITB), Universidad de La Laguna (ULL), La Laguna, Tenerife; d Department of Epidemiology and Population Health, Stanford University, Stanford; e CIBER de Enfermedades Respiratorias, Instituto de Salud Carlos III, Madrid.

**Keywords:** Bacteria, biomarker, DNA methylation, drug response, epigenetics, EWAS, microbiota, omics, personalized medicine, respiratory disease

## Abstract

Asthma is a leading worldwide biomedical concern. Patients can experience life-threatening worsening episodes (exacerbations) usually controlled by anti-inflammatory and bronchodilator drugs. However, substantial heterogeneity in treatment response exists, and a subset of patients with unresolved asthma carry the major burden of this disease. The study of the epigenome and microbiome might bridge the gap between human genetics and environmental exposure to partially explain the heterogeneity in drug response. This review aims to provide a critical examination of the existing literature on the microbiome and epigenetic studies examining associations with asthma treatments and drug response, highlight convergent pathways, address current challenges, and offer future perspectives. Current epigenetic and microbiome studies have shown the bilateral relationship between asthma pharmacologic interventions and the human epigenome and microbiome. These studies, focusing on corticosteroids and to a lesser extent on bronchodilators, azithromycin, immunotherapy, and mepolizumab, have improved the understanding of the molecular basis of treatment response and identified promising biomarkers for drug response prediction. Immune and inflammatory pathways (eg, IL-2, TNF-α, NF-κB, and C/EBPs) underlie microbiome–epigenetic associations with asthma treatment, representing potential therapeutic pathways to be targeted. A comprehensive evaluation of these omics biomarkers could significantly contribute to precision medicine and new therapeutic target discovery.

Asthma, a major biomedical concern worldwide that affects 262 million people, is the 34th leading cause of burden of disease globally and is related to 461,000 deaths every year.^1,2^ Asthma treatment, which often relies on the combination of inhaled corticosteroids (ICS) and β_2_-agonist bronchodilators, is highly effective in controlling respiratory symptoms. However, 17% of patients have difficult-to-treat asthma, and 3.7% have severe asthma that does not respond to available therapies despite adequate treatment adherence and management of modifiable risk factors.^2^ Although a small proportion of patients have severe uncontrolled disease, it disproportionately accounts for up to 50% of total asthma-related health care costs.^3^

Asthma encompasses a wide range of heterogeneous phenotypes sharing clinical characteristics, triggers, types of inflammatory processes, and distinctive treatment response profiles.^2,4^ Large differences in treatment response are driven by host factors and environmental exposure. Precision medicine has arisen to tailor preventive measures and medical treatment to obtain optimal clinical outcomes for each patient.^5^ Newer omics strategies have become essential in identifying molecular-level subtypes of the disease (ie, endotypes) to target specific therapeutic pathways.^4,5^ Indeed, biological therapies are promising asthma drugs to resolve the disease in patients with type 2 (T2)-high endotypes. However, the disease of 50% of adult asthma patients remains refractory to these therapies,^6^ requiring the discovery of novel drug-response biomarkers.

Multiple genetic loci mostly implicated in immune and airway pathologic pathways have been associated with asthma and drug response (Fig 1).^7^ However, the contribution of identified genetic and pharmacogenetic variants is small, representing only a minor fraction of heritability estimates.^7^ Given that environmental factors (tobacco smoke, air pollution, allergens, or microbial exposure) are relevant contributors to asthma susceptibility and severity,^8^ the missing asthma heritability might be driven by unaccounted gene–environment interactions. The study of the human epigenome and microbiome has attracted substantial interest because these might bridge the gap between genetic susceptibility and environmental exposure for asthma risk and drug-response variability.^7–9^

The role of the epigenome and microbiome on asthma susceptibility has been recently reviewed,^8,9^ but its implications on asthma treatment have not been discussed. Here we aim to provide a comprehensive review of the current literature on the bacterial microbiome and epigenetic studies examining associations with asthma pharmacologic interventions and drug response. We integrate convergent pathways identified through different omics approaches while addressing current gaps in the literature and offering future perspectives. The methodology of the literature search is described in Box 1 and Fig E1 (in the Online Repository available at www.jacionline.org), and the main findings are summarized in Fig 2.

## PHARMACOEPIGENETIC STUDIES IN ASTHMA

**FIG 3.**
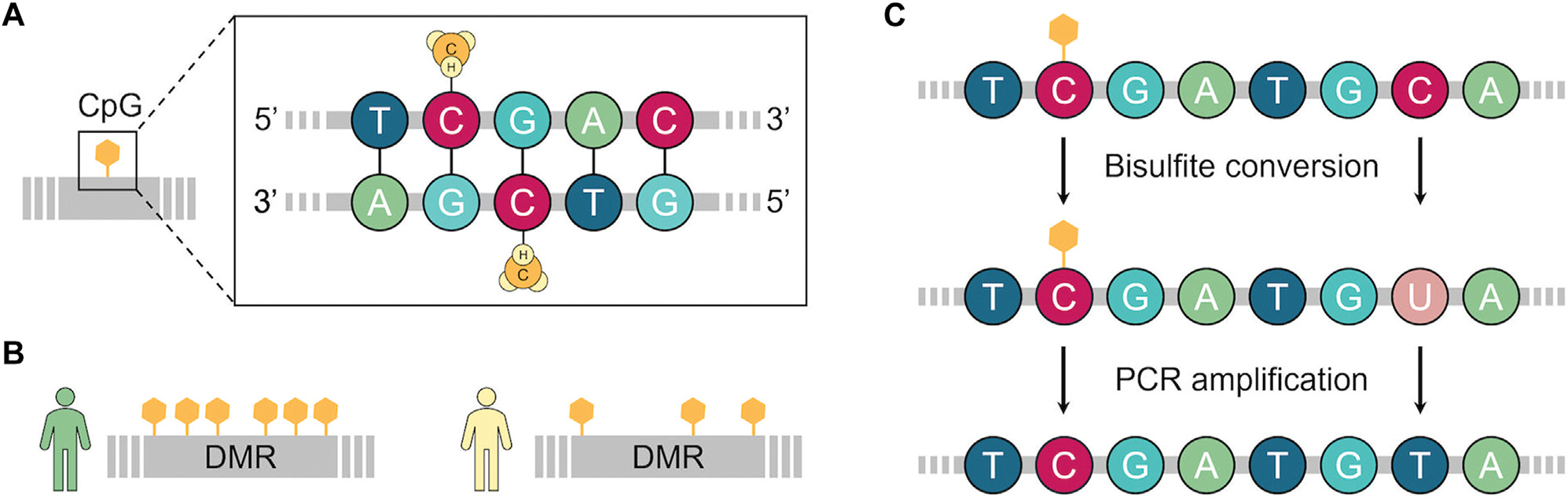
Summary of key concepts of epigenetic association studies. **A,** Representation of addition of methyl group to cytosine located at CpG site. **B,** Multiple comethylated CpGs can constitute a DMR when their DNAm patterns are distinct among different phenotypes. **C,** Main strategy to study DNAm involves initial step of addition of sodium bisulfite that leads to deamination of unmethylated cytosines to uracil, which is subsequently converted to thymine via PCR amplification. During this process, methylated cytosines remain unconverted, and standard genotyping or sequencing methods can be used to distinguish methylation status.^8^

Epigenetic mechanisms regulate gene expression without modifying the DNA sequence. DNA methylation (DNAm), consisting of the addition of a methyl group to cytosine nucleotides often located in CpG (5′-cytosine-phosphate-guanine-3′ dinucleotide) sites, is the most studied epigenetic marker in asthma.^8,10^ Unmethylated cytosines are susceptible to be deaminated and converted to uracil when treated with bisulfite sodium while methylated cytosines remain unconverted. Thus, the most commonly used techniques to measure DNAm often rely on bisulfite conversion followed by genotyping methods.^8^ DNAm can be assessed by candidate-gene or epigenome-wide association study (EWAS) approaches, the latter through microarray-based or whole-genome bisulfite sequencing, to identify CpGs and differentially methylated regions (DMRs) associated with a specific phenotype (Fig 3).^8,10^

DNAm from blood and nasal samples have been the most studied in the asthma field.^8,11^ While blood DNAm markers are expected to capture the immune and inflammatory systemic mechanisms, nasal samples may often provide complementary information about environmental exposure and the airway pathophysiology in asthma.^11^ However, adjustment for cell-tissue heterogeneity is critical when analyzing these complex tissues because epigenetic associations could reflect differences in cell-type composition instead of true epigenetic differences.^8,10^

Epigenomic studies can leverage the interaction of the epigenome with genetic variation and gene expression to identify molecular mechanisms underlying asthma pathogenesis throughout the life course. For example, asthma-associated DNAm markers might be implicated in methylation quantitative trait loci (meQTL), defined as genetic variants that partially regulate DNAm in epigenetic loci in *cis* (≤1 Mb) or in *trans* (>1 Mb).^11^ Similarly, DNAm patterns regulating gene expression, known as expression quantitative trait methylation (eQTM), can influence asthma by affecting genes involved in inflammatory and immune response, lung function, and/or transcription factors activity.^11^

A bilateral relationship has been described between DNAm and asthma pharmacologic interventions, with drug exposure inducing DNAm changes and DNAm patterns affecting treatment response. However, epigenetic studies related to pharmacologic interventions in asthma are relatively scarce, often limited to pediatric cohorts (87%), and mostly centered on corticosteroids (53%). The main characteristics and findings of these studies are described in Table I, while summary statistics and annotation of key associated CpGs and DMRs are reported in Tables E1 and E2, respectively, in the Online Repository available at www.jacionline.org.

### Glucocorticosteroids

Glucocorticosteroids can induce DNAm changes in genes involved in their anti-inflammatory or immune-modulating properties and their adverse effects, as observed in patients with chronic obstructive pulmonary disease.^12,13^ In asthma, studies suggest that epigenetic modifications induced by glucocorticosteroids might depend on the route of administration. Kere et al^14^ examined the effects of ICS on the whole-blood methylome of asthmatic children but no robust effect with evidence of replication was observed, which could be related to the limited impact of ICS on this tissue. Similarly, Yang et al^15^ found no effect of nasal corticosteroid treatment on the nasal DNAm patterns of 100 candidate epigenetic markers involved in atopic asthma.

In contrast, nasal DNAm levels of certain genes (*VNN1* and *OTX2*) might be affected by add-on systemic corticosteroid therapies in a drug-response–specific manner.^16,17^ Xiao et al^16^ reported that *VNN1* nasal expression was increased among those whose disease responded to corticosteroid therapy as a consequence of hypermethylation in the promoter region in response to corticosteroid exposure. Additionally, *VNN1*-knockout mice showed reduced airway responsiveness but also increased steroid resistance, suggesting a dual role of this gene in asthma pathogenesis.^16^ In a pilot study, Zhang et al^17^ reported hypermethylation of the *OTX2* promoter in nasal epithelia from individuals with good response to corticosteroids compared to those with a poor response. However, this locus showed differential hypomethylation in response to systemic-corticosteroid exposure only among individuals with a good response. The role of *OTX2* in asthma could be mediated by its interaction with FOXA2, a transcription factor involved in T_H_2 inflammation and lung immune defense.^18^ Additionally, 5 CpGs on *LDHC, DNHD1,* and *PRRC1* were associated with corticosteroid response, being one of them correlated with *LDHC* expression.^17^ *LDHC* encodes lactate dehydrogenase, a well-recognized biomarker for general inflammation and allergic diseases.^19^ Using epigenetic aging biomarkers, Nwanaji-Enwerem et al^20^ revisited this pilot study, reporting that nasal epigenetic age acceleration is associated with corticosteroid response in a sex-specific manner; specifically, female subjects with good response to corticosteorids showed lower epigenetic aging compared to male subjects without an adequate response. Given that nasal epigenetic aging is a useful biomarker of asthma, allergy, and lung function,^21,22^ these preliminary results suggest that epigenetic aging biomarkers might help guide asthma treatment.

The association between whole-blood methylome and response to ICS has been examined by 2 EWAS conducted by Wang et al.^23,24^ In the first study, 2 CpGs implicated as eQTM of *IL12B* and *CORT* were associated with asthma exacerbations and oral corticosteroid (OCS) receipt specifically in ICS-treated children of diverse ethnicities.^23^ *IL12B* encodes a common subunit of interleukins IL-12 and IL-23, which regulate T_H_1/T_H_2 responses and airway inflammation in asthma.^25^ *CORT* encodes the cortistatin peptide, a promising target for asthma treatment through the inhibition of the proinflammatory nuclear factor kappa–light-chain enhancer of activated B cells (NF-κB) signaling pathway.^26^ In the second study, hypermethylation of a CpG on *BOLA2* was associated with improved lung function and increased expression levels after 8 weeks of ICS treatment.^24^ The role of *BOLA2* is supported by differential expression observed in severe asthma and during the course of respiratory infections.^27,28^ Moreover, hypermethylation of *OTX2* in whole blood was associated with good response to ICS,^24^ potentially replicating the *OTX2* hypermethylation observed in the upper airways from subjects with good steroid response.^17^

Of note, none of these studies evaluated the influence of genetic variation on DNAm associations with response to ICS. Slob et al^29^ attempted to identify meQTL of CpGs altered during ICS treatment in adults with chronic obstructive pulmonary disease to be then tested for association with response to ICS in pediatric asthma. A total of 44 steroid-inducible CpGs were associated with 71 meQTL, and half of them with gene expression. However, no meQTL was associated with response to ICS, suggesting that genetic–methylation interactions with clinical effects may be disease and age specific.

### Bronchodilators

The relationship between DNAm and bronchodilators has been less explored.^21,30–32^ In the only study examining albuterol-induced DNAm changes, Perez-Garcia et al^30^ reported that albuterol predominantly showed *in vitro* hypomethylation in nasal epithelial cells affecting asthma-relevant processes (eg, IL-2, TNF-α, and NF-κB pathways). Hypomethylation was replicated in genes linked to the β_2_-adrenergic receptor pathway (*CREB3L1*, *MYLK4*, and *KSR1*).^30,33^ The hitherto unknown relationship of these genes with bronchodilators is supported by the roles of family-related proteins (CREB1 and MYLK1) in asthma development, airway inflammation, muscle contraction, and regulation of cytokines such as IL-2 and TNF-α.^34–37^ Moreover, albuterol-induced hypomethylation correlated with *KSR1* and *FLNC* expression,^30^ which encode proteins that interact with primary factors involved in adverse effects to β_2_-agonists (eg, mitogen-activated protein kinase [MAPK] and β-arrestin-2).^33,38,39^

Regarding bronchodilator drug response (BDR), Cardenas et al^21^ carried out EWAS of multiple asthma traits in nasal samples of a multiethnic pediatric cohort including 12% asthmatic children. BDR was associated with 130 significant CpGs, including top-hit epigenetic markers involved in the ubiquitin–proteasome system, actin activity, and cell adhesion (*HECTD4*, *CASP16P*, *ACTR3*, *LGALS8*, and *STIM1*). BDR was not associated with epigenetic aging and showed a unique DNAm signature not overlapping with other asthma phenotypes.^21^ Perez-Garcia et al^31^ identified DNAm markers associated with BDR in an African American population (*FGL2* and *CEBPD*) and a CpG in *DNASE2* that also replicated in a Latino population acting as *cis*-eQTM of *RNASEH2A. FGL2* suppresses non-T2 proinflammatory pathways through cytokine regulation, such as IL-2,^40^ while *DNASE2* association could be linked to DNases’ ability to treat severe asthma by disaggregating neutrophil-DNA traps and reducing airway inflammation.^41^ Martin-Almeida et al^32^ reported 4 DMRs associated with BDR in pediatric moderate-to-severe asthma. DNAm markers associated with BDR in these studies were enriched for asthma-related outcomes (fractional exhaled nitric oxide) and proinflammatory cytokine pathways, such as IL-2 and TNF-α.^31,32^ Fractional exhaled nitric oxide was examined as an airway-inflammation proxy by Cardenas et al and Martin-Almeida et al, identifying DNAm markers linked to T2 inflammation, granulocyte activity, and proinflammatory factors, such as IL-2, IL-4, NF-κB, CCAAT/enhancer-binding proteins (C/EBPs).^21,32^

Supported by the impact of ancestry on QTL architecture,^42^ genetic ancestry may influence the BDR-epigenetics relationship, as some population-specific DNAm markers were associated with genetic variation, while population-shared ones were associated with environmental exposures.^31^ Moreover, Perez-Garcia et al^31^ revealed the potential clinical applicability of pharmacoepigenetics through the development of a model based on 70 CpGs that classified albuterol response similarly to clinical predictors and overcoming pharmacogenetic variants.

### Immunotherapy

The effect of dust mite–specific allergen immunotherapy (SIT) on blood DNAm was explored by Wang et al.^43,44^ Focusing on CpGs regulating cytokine genes, mite-SIT was shown to induce hypermethylation in *IL4* as a consequence of decreasing IL-2 signaling.^43^ At the epigenomic scale, mite-SIT induced DNAm changes associated with gene expression in *BCL6, HSPG2,* and *HSP90AA1*^44^—genes involved in T_H_2 inflammation, cytokine regulation, airway remodeling, and epithelial barrier dysfunction.^45–47^

### Biological therapies

Rakkar et al^48^ evaluated DNAm and gene expression changes in nasal brushings of adult asthma patients treated with mepolizumab. A total of 53 CpGs were differentially methylated after 3 months of mepolizumab treatment, potentially mediating the transcriptomic response to mepolizumab related to cell homeostasis. Baseline DNAm was not associated with drug response, and longitudinal DNAm changes were not evident on stratification according to response and nonresponse to mepolizumab. Alternatively, transcriptomic analyses showed that individuals with a poor response may exhibit a higher degree of inflammation than those with a good response characterized by increased *IL4*, *IL33*, and *TLSP* and reduced *IL1A*, *IL1B*, and *IFNA1* expression.^48^

## MICROBIOME STUDIES OF PHARMACOLOGIC INTERVENTIONS AND DRUG RESPONSE IN ASTHMA

**FIG 4.**
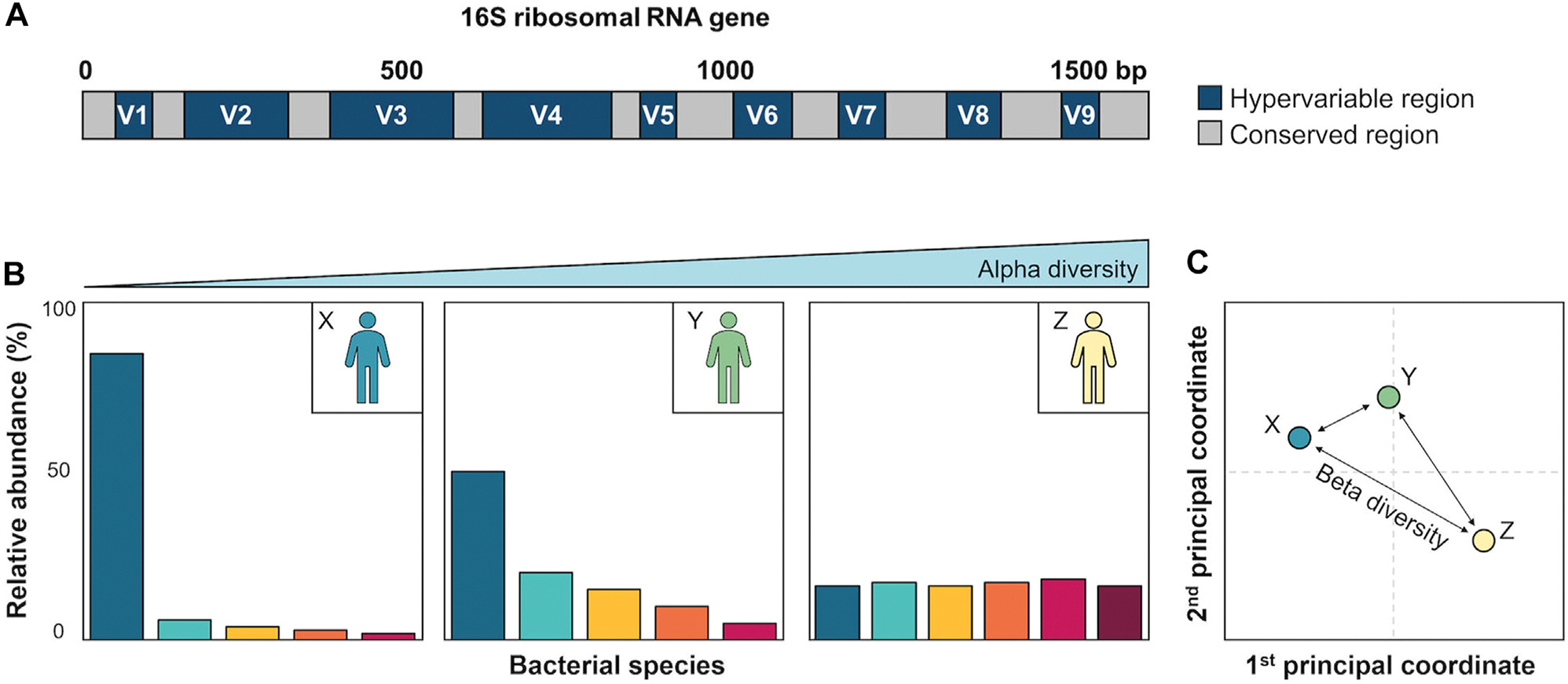
**A,** Structure of 16S ribosomal RNA gene. This locus spanning approximately 1.5 kb is characteristic of prokaryotic cells and contains 9 hypervariable regions (V1-V9) that provide detailed taxonomic information and are flanked by highly conserved regions that serve as binding sites for PCR primers.^49^ **B,** Representation of microbial communities of 3 subjects. Each bar corresponds to a bacterial species (different species are distinctly *colored*) whose relative abundance is plotted on y-axis. α-Diversity quantifies diversity of distinct bacteria within individual samples. It increases (*to the right*) proportionally with number of species observed (richness) and their even distribution. **C,** β-Diversity reflects differences in bacterial composition between samples and is often useful for visualization through principal coordinates analyses. Distances between points are proportional to dissimilarities across samples. Samples with more similar bacterial profiles (eg, X and Y) are closely visualized.

The human microbiome is the collection of genetically characterized microorganisms (eg, bacteria, fungi, viruses) inhabiting the human body. Metagenomics leads to the accurate detection of microorganisms (species or strain levels) and their whole-genome annotation.^49^ Nevertheless, targeted sequencing of the 16S ribosomal RNA gene is the most common approach used for bacterial microbiome profiling in asthma (Fig 4).^9,50^ It is a well-tested and cost-effective method for bacterial microbiome analysis at the genus level even in low-biomass samples.^49^

Microbial exposure (ie, environmental microbiota, host microbiome, and respiratory infections) are one of the main mechanisms underlying gene–environment interactions in asthma.^9,50^ The microbiome, especially the airway and gut microbiomes, affect asthma by modulating and training the immune system and contributing directly to airway injury.^9,50^ Indeed, airway microbes may induce lung injury, epithelial barrier dysfunction, and T_H_2 inflammation, while the gut microbiome has a key role in host immunity and is linked to airway health through the gut–lung axis.^9,50^ Although environmental factors substantially contribute to the microbiome, some bacteria are heritable, and host genetics can modulate their influence on asthma.^50,51^ Microbiome genome-wide association studies (mbGWAS) have allowed the identification at genomic scale of host genetic variants influencing the microbiome composition, known as microbiome quantitative trait loci (mbQTL).^51^

Similar to epigenetics, research to date has shown the bilateral relationship between the human microbiome and asthma pharmacologic interventions. Studies have almost exclusively profiled the respiratory microbiome (90%), predominantly in adult asthma patients (72%), and focusing on corticosteroids (70%) (Table II).

### Glucocorticosteroids

Corticosteroid-induced microbial changes have been primarily evaluated on the lung and sputum microbiomes,^52–60^ suggested to be mediated by their immunosuppressive effect, disturbance of the mucosal immune system, and overgrowth of certain bacteria in the airways.^61^ Huang et al^54^ observed that Actinobacteria and Gammaproteobacteria (eg, *Klebsiella*) were enriched in the bronchial microbiome of patients with severe asthma treated with high-dose ICS, while Proteobacteria were increased in mild-to-moderate asthma patients treated at lower doses. Actinobacteria and bacterial α-diversity were positively correlated with *FKBP5* expression,^54^ a steroid-response marker thought to underlie corticosteroid mechanisms of action.^62^ Denner et al^55^ reported that ICS alter the bronchial microbiome (decreased *Veillonella* and *Prevotella*), being this dysbiosis expanded by add-on OCS treatment (ie, changes in *Rickettsia*, *Pseudomonas*, *Lactobacillus*, and *Streptococcus* abundances).

Martin et al^59^ examined the sputum microbiome in patients with clinically stable asthma. Bacterial communities were stable after 14 days of ICS treatment, but *Streptococcus* and *Prevotella* abundances increased in patients treated with low-dose ICS, while *Dysgonomonas* and *Parvimonas* were more abundant in the high-dose group. High-dose fluticasone was associated with increased *Haemophilus parainfluenzae* compared to budesonide,^59^ an opportunistic pathogen associated with steroid resistance^63^ potentially related to the higher pneumonia risk in fluticasone-treated asthma patients.^64^

The impact of glucocorticosteroids on the interactions between bacteria and fungi has also been explored.^52,53,60^ Huang et al^52^ found that ICS treatment may reduce the overgrowth of potential pathogenic bacteria related to eosinophil levels (eg, *Streptococcus*, *Gemella*, and *Neisseria*) enriched in the bronchial microbiome from untreated asthma patients. In contrast, a 9-month longitudinal study showed no effect of ICS on the bronchial microbiome.^53^ Both studies supported that disruption of bacteria–fungi interactions contributes to asthma development; ICS may not reverse this disruption but could diminish it.^52,53^

Focusing on the lung microbiome, Sharma et al^60^ observed that ICS treatment reduced atopy-related bacteria (*Stenotrophomonas* and *Atopobium*), whereas OCS decreased bacteria enriched in T2-low asthma patients (*Actinomyces*). Corticosteroid-induced disruptions affected bacterial pathways involved in allergic and inflammatory respiratory diseases, such as ATP-binding cassette transporters, bacterial kinases, and ubiquitin proteasome, by altering the NF-κB, IL-6, TNF-α, and IFN-γ pathways.^65–67^ Notably, Huang et al and Sharma et al converged on ICS-induced depletion of potential fungal pathogens and their interaction with other microorganisms involved in asthma development (eg, *Penicillium* and *Aspergillus*).^52,53,60^

By using a shotgun metagenomic approach in a 6-week longitudinal study of ICS treatment, Turturice et al^58^ identified that ICS increased bacterial α-diversity and reduced the abundance of *Enterococcus faecium* and *E faecalis* and upper airway pathogenic species (*Streptococcus pneumoniae* and *Neisseria meningitidis*) in the lung microbiome. These bacteria were associated with cytokines involved in the ICS-induced improvement of lung function, such as IL-2, TNF-α, and chemokine C-C motif ligand 4 (CCL4).^58^ However, inflammatory biomarkers and microbiome–cytokine interactions diminished after ICS treatment, supporting a relationship between lung microbiome, cytokine profile, and response to ICS in asthma.^58^

Durack et al^56,57^ showed that ICS microbiome disruptions may be drug-response specific. Given that airway microbiome differed between those with good response to ICS (increased Streptococcaceae, Fusobacteriaceae, and Sphingomonadaceae) and those with a poor response (increased Pasteurellaceae, such as *Haemophilus*), the authors prospectively evaluated the effect of 6 weeks’ ICS treatment in both groups.^56,57^ While ICS altered the bronchial microbiome composition in those with good response to ICS (ie, increased *Neisseria, Moraxella,* and Microbacteriaceae, and reduced *Fusobacterium*),^56^ higher alterations in β-diversity and composition of sputum microbiome were observed in those with a poor response.^57^ These distinct perturbations might be related to enhanced xenobiotic biodegradation of bacteria present in the airways of those without an adequate response.^56^

The upper airway microbiome has also been linked with the effectiveness of ICS in preventing asthma exacerbations. Zhou et al^68^ revealed that the dominance of the nasal microbiome by *Corynebacterium* and *Dolosigranulum* reduced the risk of asthma exacerbations in ICS-treated children. Conversely, during asthma exacerbations, the airway microbiome tends to be dominated by potentially pathogenic bacteria related to asthma exacerbations and respiratory infections (*Streptococcus* and *Moraxella*).^68,69^ Perez-Garcia et al^70^ reported 18 bacterial genera in the salivary, nasal, and pharyngeal microbiome associated with lower risks of asthma exacerbations despite ICS treatment. These included promising therapeutic probiotics that reduce airway hyperreactivity, inhibit NF-κB-mediated inflammation, and enhance ICS therapeutic effects (eg, *Bifidobacterium*, *Selenomonas*, *Porphyromonas*, and *Rothia*).^71–74^ These studies supported the protective effect of higher bacterial α-diversity against asthma development and exacerbations.^68,70,75^ Notably, the salivary microbiome was proposed as a noninvasive source of bacterial biomarkers with clinical applicability that improves predictive models of asthma exacerbations.^70^

Moreover, the lung microbiome was shown to influence OCS response by Goleva et al.^63,76^ Gram-negative bacteria with high endotoxic activity, such as *Haemophilus*, might participate in steroid unresponsiveness via MAPK and IL-8 pathways, while long acyl chain lipid A producers with low endotoxicity, such as *Fusobacterium*, might increase the sensitivity to these drugs.^63^ In addition, receipt of acid suppression drugs, which tends to be higher among patients with steroid-resistant disease, can lead to the expansion of opportunistic pathogens of the *Streptococcus mitis* group (*S mitis* and *S pseudopneumoniae*).^76^ These pathogens reduced *in vitro* the response to dexamethasone via p38 MAPK in bronchial epithelia compared to commensal *Streptococcus oralis,* likely related to the virulence factors produced by those species.^76^

Finally, host–microbiome interactions might also have significant implications for drug response. Perez-Garcia et al^77^ conducted the first mbGWAS in asthma, reporting that genes associated with ICS-response–related bacteria participate in the development of asthma comorbidities closely related to refractory asthma and microbiome dysbioses—for example, obesity, tobacco use disorder, and reflux esophagitis.^78–80^ Additionally, potential drugs and therapeutic targets for asthma exacerbations likely regulating these genes were highlighted, which were involved in inflammation and corticosteroid response (ie, trichostatin A, NF-κB, glucocorticosteroid receptor, and C/EBPs).^62,77,81^ Moreover, 3 genetic loci previously associated with response to ICS (*APOBEC3B-APOBEC3C*, *TRIM24*, and *TPST2*) were implicated as mbQTL of exacerbation-related airway bacteria, supporting a genetic–microbiome–ICS–response relationship.^77^

### Azithromycin

Slater et al^82^ provided preliminary evidence of azithromycin-induced lung microbiome alterations by decreasing potential pathogenic bacteria abundance. However, Wei et al^83^ and Park et al^84^ supported nonperdurable dysbiosis in the gut rather than in the lungs. Azithromycin alleviated airway inflammation in mice by increasing the gut abundance of short-chain fatty acid (SCFA) producers, such as Clostridiales.^84^ SCFAs diminish eosinophil activity, airway hyperresponsiveness, and T2-cytokine production in lung T cells.^84,85^ Nonetheless, azithromycin reduced bacterial α-diversity and early-life commensals (*Lactobacillus* and *Bifidobacterium*) during gut-microbiome maturation.^83,84^ This may have implications on probiotic efficacy during the life-span because bacteria involved in asthma onset and allergic-persistent inflammation may be distinct.^84^

Similarly, Lopes Dos Santos Santiago et al^86^ described a nonperdurable dysbiosis of the oropharyngeal microbiome (increased *Streptococcus salivarius* and decreased *Leptotrichia wadei* abundances) after a 6-month azithromycin treatment. Thorsen et al^87^ reported that therapeutic response to azithromycin in asthma was linked to higher basal microbiome richness, increased *Veillonella, Leuconostoc,* and *Vibrio,* and decreased *Neisseria.*^87^ Azithromycin’s effectiveness relies on its antiviral and anti-inflammatory properties, with the antibacterial effect playing a key role in treating children who experience an inappropriate antibacterial immune response (low TNF-α and IL-10 and high CCL22).^87,88^

### Biological therapies

Diver et al^89^ examined the effect of mepolizumab on the microbiome of 13 asthma patients. This preliminary study showed no microbial dysbiosis after 12 weeks’ treatment, but it may not be conclusive because of the limited sample size and relatively short study period. Indeed, other biological drugs used for allergies have therapeutic effects on recovering microbiome dysbiosis (eg, dupilumab)^90^ or increase the risk of airway-infection–triggered asthma exacerbations due to rapid eosinophil depletion (eg, benralizumab).^91^

## COMMON FINDINGS OF EPIGENETICS AND MICROBIOME STUDIES OF ASTHMA TREATMENT

Epigenetic modifications are being recognized as mediators of host–microbiome interactions in human physiology.^92,93^ Environmental stimuli from the microbiome and bacteria-produced metabolites can affect the epigenetic landscape by different mechanisms, such as the microbial biosynthesis of chemical donors required for DNAm (eg, *S*-adenosylmethionine), the regulation of epigenetic-modifying enzymes (inhibition of histone deacetylases, known as HDACs, through SCFA production), or the activation of host-cell intrinsic processes leading to epigenetic modifications.^93^ Microbial exposure, especially *in utero* and during early life, promotes, through epigenetic modifications, a functionally trained immunity programming that influences the immune responses to postnatal exposure.^92^ Thus, perturbations in early-life microbial exposure can contribute to asthma and allergy development in later life and can be reflected in persistent epigenetic modifications.^50,94^

**FIG 5.**
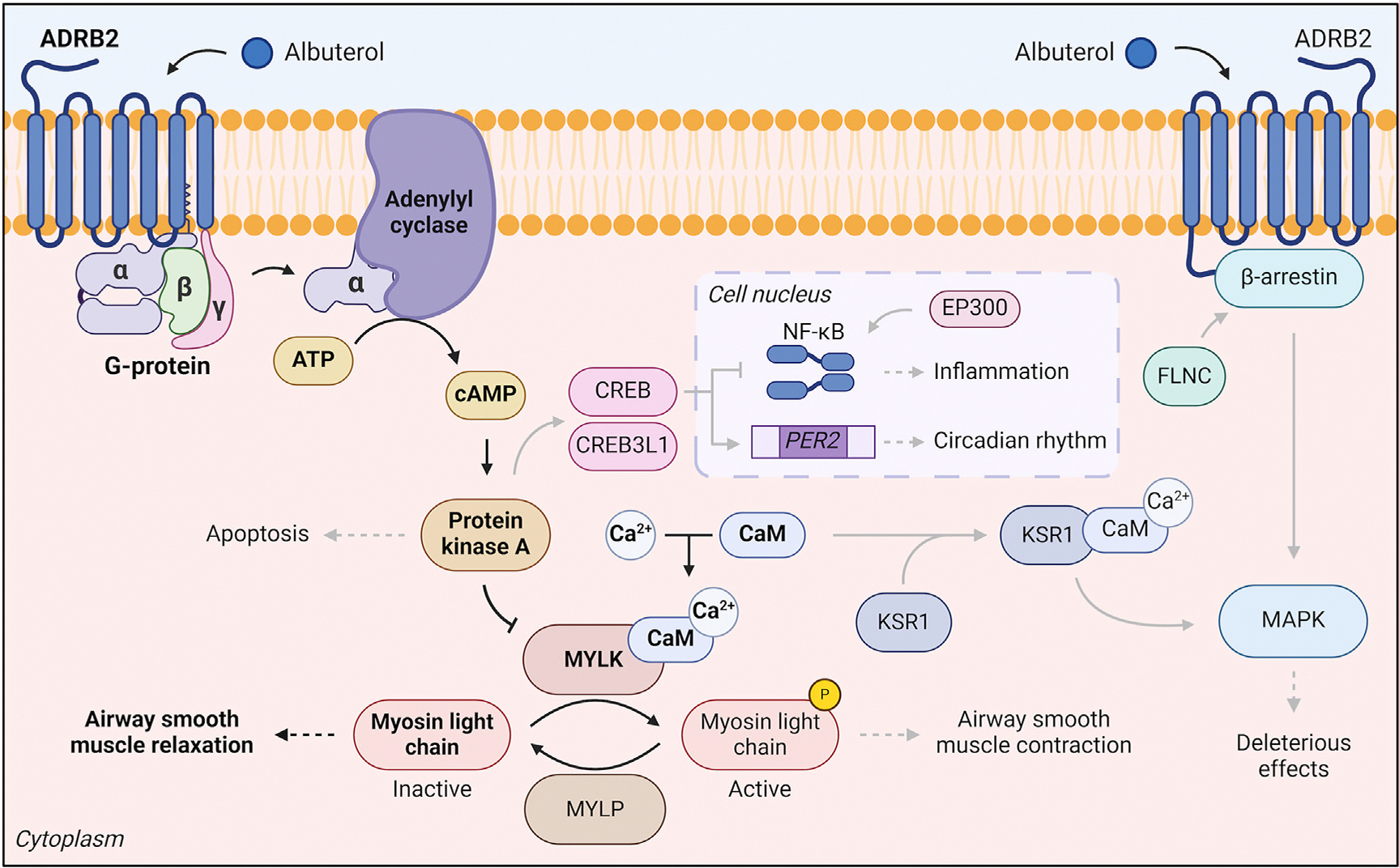
Main mechanism of action of albuterol via adrenergic β_2_ receptor (ADRB2) signaling pathway *(black arrows and boldface)*^33^ and potential factors hypothesized to underlie biological effects of albuterol *(gray arrows). Arrows* indicate molecular interactions/relations *(sharp),* inhibition *(blunt),* and biological effects *(dashed).* Routes involving multiple mediators are simplified for representation. Airway smooth muscle tone is regulated by phosphorylation status of myosin light chains through myosin light chain kinases (MYLK) and phosphatases (MYLP). Albuterol binding to ADRB2 initiates a signal cascade via cyclic adenylate monophosphate (cAMP) and protein kinase A (PKA) that inhibits myosin light chains phosphorylation and leads to bronchodilation. This pathway can be blocked by β-arrestin-2, responsible for albuterol’s main side effects through the MAPK pathway.^33^ Potential factors and routes hypothesized to underlie albuterol’s biological effects based on differentially methylated loci after albuterol exposure in airway epithelia include processes such as regulation of inflammation, bronchodilation, apoptosis, or circadian rhythm. Further details are reported in the Online Repository.^30^ Created with BioRender.com.

**FIG 6.**
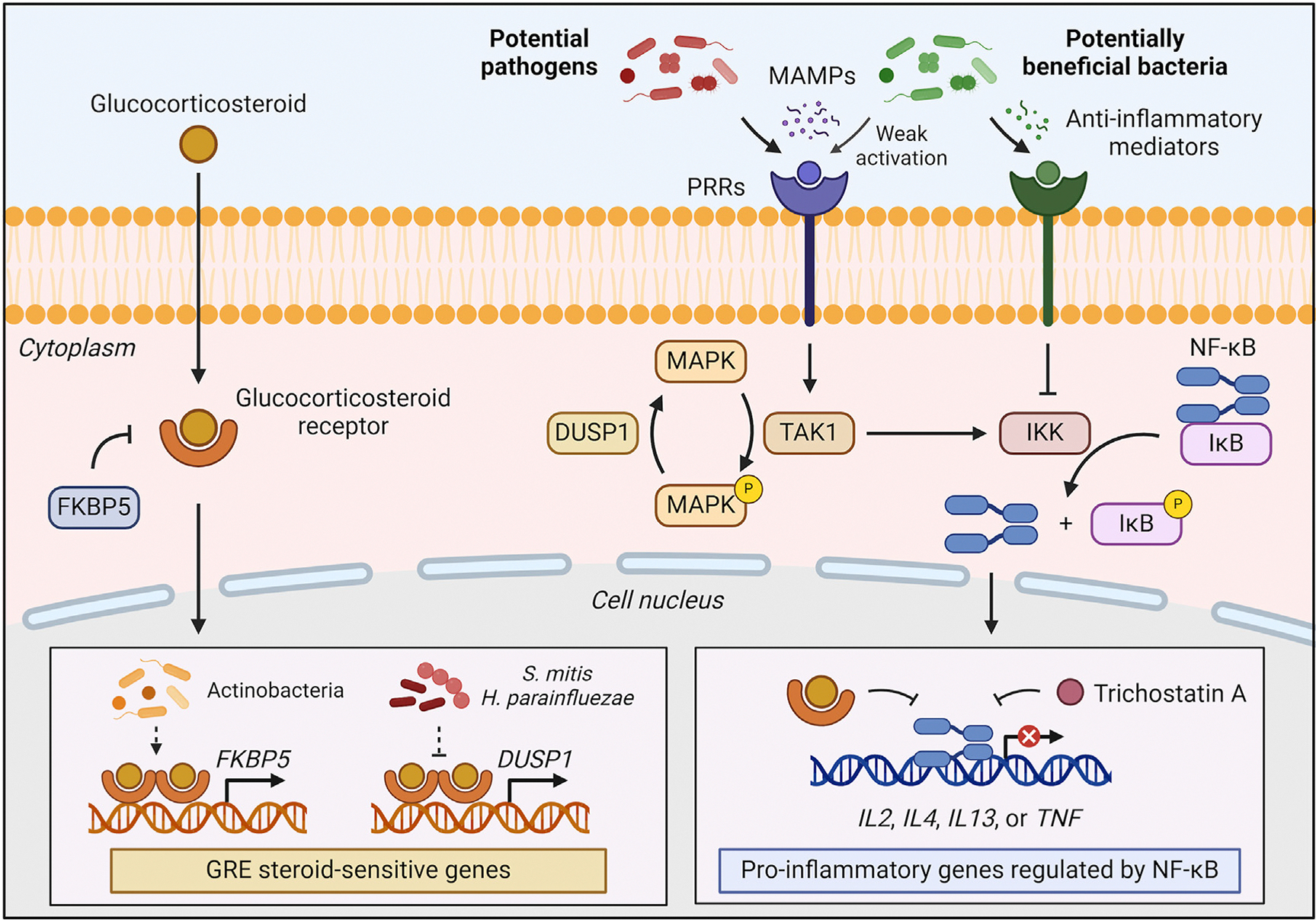
Main mechanism of action of glucocorticosteroids and potential influence of microbial exposures. *Arrows* indicate molecular interactions *(sharp),* inhibition *(blunt),* or undefined pathways *(dashed).* Glucocorticosteroids diffuse across cell membrane and bind to glucocorticosteroid receptor α-isoform (GR).^13^ In nucleus, GR homodimers bind to glucocorticoid response elements (GREs) in promoters to directly or indirectly regulate steroid-sensitive genes. Glucocorticosteroids silence proinflammatory genes activated by the MAPK pathway and NF-κB,^13^ a transcription factor also inhibited by trichostatin A (a potential drug for asthma exacerbation treatment).^77,81,96^ Bacterial pathogens may promote corticosteroid resistance by activating proinflammatory pathways (such as MAPK), inducing genes that reduce GR sensitivity (*FKBP5*), or hindering anti-inflammatory gene activation (*DUSP1*).^54,63,76^ Microbial-associated molecular patterns (MAMPs), recognized by pattern-recognition receptors (PRRs), activate transforming growth factor β–activated kinase 1 (TAK1). It leads to activation of NF-κB, through inhibitor of kappa B (IκB) phosphorylation by IκB kinase (IKK), and MAPK pathway.^63^ Alternatively, low-endotoxin bacteria (*Prevotella melaninogenica*) weakly activate proinflammatory pathways,^63^ while beneficial bacteria (*Bifidobacterium* spp or *Rothia mucilaginosa*) enhance corticosteroid response through NF-κB inhibition or induction of host anti-inflammatory mediators.^70,71,74^ Specific bacterial mediators involved remained undetermined. Created with BioRender.com.

Although host epigenetic–microbial interactions remain unexplored in the context of asthma treatment, discoveries from single-omic studies suggest that methylation markers and bacterial exposure are potentially implicated in the mechanism of action of main asthma drugs, such as bronchodilators (Fig 5) and corticosteroids (Fig 6). Additionally, epigenetic and microbiome studies converge on the potential implication of inflammatory and immune pathways, especially those involving IL-2, IL-4, TNF-α, NF-κB, and C/EBPs, on the relationship between the microbiome, epigenetics, and asthma pharmacologic interventions.^23,30–32,43,58,60,70,77,87^ Given that anti–IL-4R therapies are showing benefits in treating severe T2-high asthma,^6^ further evaluation of these pathways could provide the identification of promising target candidates for asthma treatment.

NF-κB is a key proinflammatory transcription factor that promotes airway and allergic inflammation, and its continued activation potentially underlies the persistent airway inflammation observed in severe asthma.^62,95^ It acts as a link between asthma and microbial exposure.^62^ While exacerbation-triggering pathogens enhance the NF-κB pathway, commensal bacteria alleviating asthma symptoms inhibit this pathway.^71,74^ The NF-κB pathway participates in the bilateral relationship between the epigenome and microbiome with ICS and bronchodilators,^23,30,32,60,70,77^ being a potential therapeutic target for respiratory diseases. Glucocorticosteroids are the main NF-κB inhibitors, but newer therapies targeting specific pathway-involved molecules are under evaluation in clinical trials.^62,95^ Notably, genomic, epigenomic, and microbiome studies of asthma therapies have suggested trichostatin A, an inhibitor of the NF-κB pathway, as a potential drug for asthma treatment, but further research on its efficacy and safety is needed.^30,77,81,96^

C/EBPs, including C/EBPα to C/EBPζ isoforms, are transcription factors influencing the development of lung diseases and drug responsiveness.^97^ C/EBPα, crucial for maintaining lung integrity, is often deficient in airway smooth muscle cells from asthma patients, leading to airway remodeling and steroid resistance.^97^ C/EBPs are regulated by glucocorticosteroids and β_2_-agonists and mediate the agonistic mechanisms between these drugs, including NF-κB inhibition.^97^ Ongoing mechanistic studies are focusing on C/EBP members, such as *CEBPD,* which is a glucocorticosteroid-regulated gene implicated in fetal lung development.^62^ However, to date, no asthma therapy targeting C/EBPs exists. Elucidating the detected relationship between DNAm and microbiome biomarkers with C/EBPs and asthma treatment^31,77^ may provide the discovery of potential therapeutic targets involved in this pathway.

Finally, epigenetic and microbial mechanisms have been shown to participate in the implications of IL-2 and TNF-α pathways in asthma therapy.^30–32,43,58,60,87^ IL-2 and TNF-α cytokines are candidate targets for asthma treatment, given their role in allergies.^98^ IL-2 is involved in the regulation of immune cells and T_H_2 inflammation, and TNF-α plays key roles in host defense and inflammation homeostasis.^98^ However, to date, targeting these pathways for asthma treatment has not demonstrated an adequate benefit–risk balance. IL-2–based therapies have beneficial effects through regulatory T-cell activation, but IL-2 dysregulation may result in harmful and severe toxicities.^99^ Nonetheless, newer IL-2/anti–IL-2 complexes have shown promising results in reestablishing immune tolerance in asthma without harmful effects in murine models.^99^ Similarly, although no strong evidence supports TNF-α inhibitors for asthma treatment, TNF-α–dependent inflammatory memory represents a target for asthma therapy.^100^

## CURRENT GAPS AND FUTURE PERSPECTIVES

The human microbiome and epigenome are of great clinical interest for asthma treatment because they can be modifiable or used for therapeutic purposes—for example, through DNAm inhibitors, CRISPR/dCas9 systems, or bacterial lysates, probiotics, prebiotics, postbiotics, bacteriophages, and bacterial monoclonal therapies (Box 2).^71,74,101–104^ These personalized therapies are promising in reducing inflammation and/or allergic biomarkers, but there is still little evidence for a clinical application in the context of asthma. However, microbiome modulation through different techniques, such as fecal microbiota transplantation, has been proven to augment therapeutic responses in other diseases, like cancer, by its interplay with drug pharmacokinetics and/or pharmacodynamics.^105^

Despite advances provided by epigenetic and microbiome studies of asthma pharmacologic interventions, additional research is needed to overcome current limitations (small sample sizes, lack of replication, and generalizability across diverse populations) and address unanswered issues in the field. Given the dynamism of the epigenome and microbiome, cross-sectional studies have not provided information about causality. It remains unclear whether some microbiome and epigenetic associations are the consequence rather than the cause of treatment unresponsiveness. Furthermore, such fluctuation may lead to low-stability epigenetic markers and microbiome changes not being detected in observational studies, despite being potentially relevant to treatment response. Subsequent studies should leverage longitudinal approaches with detailed asthma phenotypes to overcome this issue while increasing the statistical power. Otherwise, alternative statistical approaches, such as Mendelian randomization, should be considered.

Future microbiome research might benefit from shotgun metagenomics and metatranscriptomics to identify the bacterial species and strains underlying the associations, their functionality, and their interactions with other microorganisms, such as fungi and viruses.^10,49^ Additionally, combining sequencing data with novel living-discriminating microbial composition tools would provide insights into the living bacterial microenvironment with higher potential implications in phenotype development.^10^ Furthermore, although developing microbiome-targeted therapies for asthma relies on a first discovery step, verifying the properties of candidate microorganisms, exploring the specific mechanisms, and validating their influence on clinical outcomes are required for probiotics development.^101^

Despite the economic and technical benefits of microarrays, >97% of CpGs in the human epigenome remain unexplored with the current available ones,^8^ limiting the study to methylation markers that may or may not be informative to the phenotype of interest. Recent efforts have been made to design cost–benefit custom arrays covering potentially high-value CpGs in asthma and allergies from a functional perspective.^106^ Although their use is currently constrained by their high costs, bisulfite-sequencing tools for in-depth profiling of unexplored epigenetic variation and regulatory elements are crucial for gaining further insights into drug response.^10^ Additionally, cell-type deconvolution and single-cell sequencing approaches are key to decipher specific cell types driving drug–response associations and identify novel associations nondetected when analyzing tissue-level bulk DNAm data. Furthermore, also applicable to microbiome studies, better characterization of environmental exposure will allow an understanding of the poorly studied influence of (epi)genetic–microbiome–environment interactions on asthma treatment response.

Upcoming studies should also take into consideration asthma’s large heterogeneity.^2,4^ Different asthma subtypes (eg, allergic *vs* nonallergic asthma) have demonstrated shared and distinctive genetic architectures.^4^ Each asthma subtype can exhibit different therapeutic responses,^2,4^ highlighting that molecular mechanisms underlying treatment response might be diverse. Although conducting joint or cross-trait analyses of asthma subtypes allows the identification of shared mechanisms, minimizing asthma heterogeneity in observational studies, at the cost of reducing sample size, would improve the statistical power to identify subtype-specific biomarkers with potential application for precision medicine.^4,107^

Furthermore, as previously discussed, epigenetic regulators and microbial exposure during early life play key roles in the training of innate immunity (Toll-like receptor and NF-κB pathways), IgE regulation (FceR pathway), cytokine production (IFN-γ, TNF-α, and several interleukins), and immune cells activity and proliferation (eosinophils, neutrophils, and T-cell subpopulations).^9,108–110^ Exploring how epigenetic regulators and microbiome changes exhibited during childhood persist into adulthood will provide useful insights into discovering biomarkers of treatment response in these 2 well-differentiated asthma groups. Indeed, it has been documented that probiotics considered potentially advantageous in children by training the immune system may not yield the same benefits in adults, and *vice versa.*^84^ Current DNAm and microbiome studies of asthma treatment response are highly unbalanced in the inclusion of different age groups (Tables I and II). Although few studies that jointly analyzed pediatric and adult populations detected common associations,^29,30,70,77^ specifically designed comparative studies are necessary to draw robust conclusions about the role of the epigenome and microbiome in these age groups.^4^

The study of other epigenetic biomarkers beyond DNAm will be instrumental in understanding the heterogeneity in asthma treatment response.^104^ Specifically, noncoding RNAs, including microRNAs and long noncoding RNAs, play key roles in immune function and respiratory health.^104,108–112^ Noncoding RNAs and DNAm participate in the epigenetic reprogramming that occurs during early childhood in response to risk exposure for asthma development in later life (eg, bronchiolitis),^108–110^ and they are a potential link between epigenetic and microbial exposure. MicroRNAs are implicated in response to ICS through the modulation of the proinflammatory NF-κB pathway, with a potential predictive ability of drug response.^113^ Currently, these have shown promising results in explicating the mechanisms underlying asthma subtypes, especially T2-low phenotypes, which currently lack phenotype-specific biomarkers with clinical applicability.^104,111,112^ Additionally, histone modifications represent key epigenetic markers involved in chromatin landscape modulation with a main role in the pathobiology of T cells in the context of asthma.^104,114^ Imbalances in the activity of histone-modifying enzyme, such as HDAC2, condition treatment response to different major asthma drugs like corticosteroids, combo medications, and antileukotrienes.^114–116^

Moreover, although single-omic–based biomarkers, such as methylation risk scores, are promising in predicting asthma and drug-response–related outcomes, their predictive ability is improved through multiomics integration.^31,70,117^ The clinical applicability of omics biomarkers would benefit from integrative omics systems biology approaches that would improve our understanding of the mechanisms of action of asthma drugs and therapy response.^10^ Future research would also benefit from expanding research beyond corticosteroids to other promising asthma drugs, such as biological therapies. However, these strategies will require advancing statistical methodologies, larger sample sizes, and deeply phenotyped individuals (ie, endotyping). We argue for collaborative efforts, similar to existing collaborations in genomic studies,^96^ to overcome these issues, increase the representation of genetically diverse populations, and improve the clinical translational of the results into personalized management strategies. Indeed, drug targets identified by genomic studies will be twice as likely to succeed in clinical trials than non–genetically supported drugs.^118^

As recapitulated in this review, potential epigenetic and microbiome biomarkers of interest to predict asthma drug response, likely involved in immunologic and inflammatory pathways, have been identified. However, the specific mechanisms and mediators underlying the bilateral relationship between the epigenome, the microbiome, and asthma treatment remain to be determined. Study heterogeneity—in terms of outcomes, biological samples, phenotypes, and age groups—and lack of functional studies have hampered the replication and prioritization of identified factors. The resolution of current gaps, coupled with the continuous development of increasingly sophisticated and accessible omics tools, will enable researchers to explore further the incompletely understood role of the epigenome and microbiome in asthma treatment.

## CONCLUSIONS

Recent advances in epigenetic and microbiome research have shed light on the molecular basis of asthma treatment. Promising biomarkers have emerged, highlighting the potential of pharmacoepigenetics and the airway microbiome to improve genetic and clinical predictive models of drug response. It is increasingly evident that immune and inflammatory pathways (eg, IL-2, TNF-α, NF-κB, and C/EBP) play key roles in the interplay between microbiome, epigenetics, and pharmacologic interventions in asthma. Future research will contribute to a deeper understanding of the role of epigenome and microbiome on treatment response, moving toward precision medicine in asthma through biomarker and therapeutic target discovery.

## Supplementary Material

Supplementary Tables E1 and E2

Supplemental text and Fig E1

## Figures and Tables

**FIG 1.**
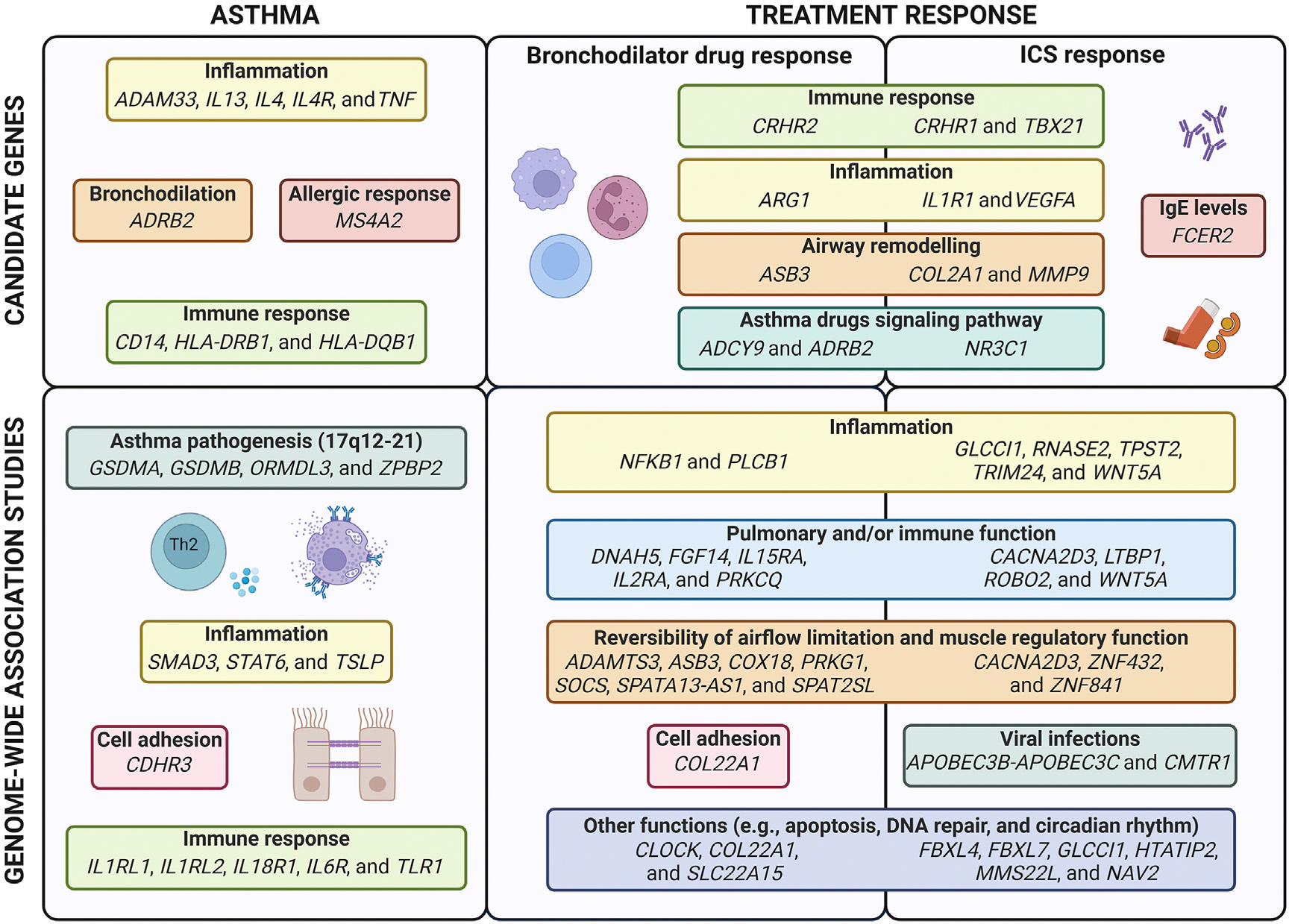
Schematic representation of top genes associated with asthma susceptibility *(left)* and treatment response *(right)* by candidate-gene approaches *(top)* and genome-wide association studies *(bottom).* Only main genetic associations with response to major asthma therapies (ICS, bronchodilators) are represented. Created with BioRender.com.

**FIG 2.**
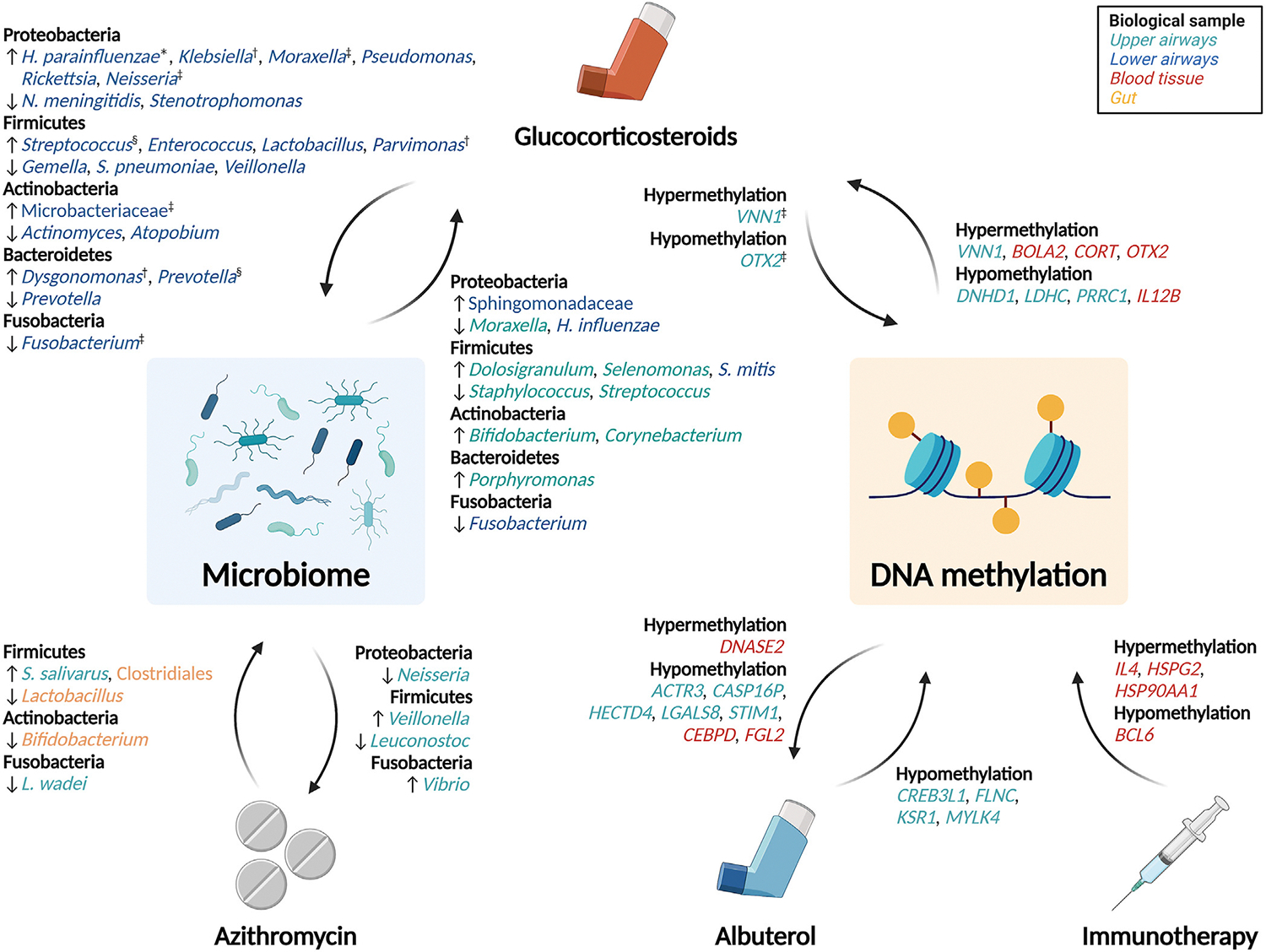
Main findings of bilateral relationship between the human microbiome and the epigenome with asthma therapies. *Arrows pointing from therapies* indicate changes in the microbiome or the epigenome related to exposure to each treatment. *Arrows pointing toward therapies* indicate epigenetic markers and bacteria associated with treatment response. Positive associations of bacteria taxa and epigenetic loci with drug exposure or treatment response are indicated with ↑ symbol and hypermethylation; negative associations are represented with ↓ symbol and hypomethylation. Bacteria taxa and genes are *colored* according to type of biological sample where they were discovered. *In patients treated with fluticasone. †In patients treated with high doses. ‡In those with good response to corticosteroids. ^§^In patients treated with low doses. Created with BioRender.com.

**TABLE I.** Summary of pharmacoepigenetic studies in asthma published until April 2024

Study	Year	Approach	Therapy	Outcome	Sample size	Age group	Ancestry	Biological sample	Main findings

Xiao et al^16^	2015	Candidate gene	SCS, ICS	Effects on DNAm and drug response	18	5–18 y	EUR 11.1%, Other 88.9%	Nasal brushings	One CpG at *VNN1* promoter region was hypermethylated by GC treatment and associated with response to this medication (hospital discharge in ≤24 h) and *VNN1* expression.
Yang et al^15^	2017	EWAS (450K array)	NCS	Effects on DNAm	36	10–12 y	AA 91.7%, LAT 8.3%	Nasal brushings	Among 118 of 119 CpGs/DMRs associated with allergic asthma, none was associated with NCS treatment.
Zhang et al^17^	2017	EWAS (450K array)	SCS, ICS	Effects on DNAm and drug response	Discovery, 20 (longitudinal)	5–18 y	Discovery, AFR 70%, EUR 20%, MIX 10%	Nasal brushings	SCS treatment induced DNAm changes in promoter region of *OTX2* in those with a good response. CpGs at *DNHD1, LDHC*, and *PRRC1* were associated with treatment response (hospital discharge in ≤24 h).
					Pyrosequencing validation, 33 (including discovery subjects)		Validation, AFR 77%, EUR 15%, MIX 8%		
Wang et al^23^	2019	EWAS (27K array)	ICS	Drug response	Discovery, 154	5–12 y	Discovery, EUR	Whole blood	DNAm in 14 CpGs, including CpGs annotated to *IL12B* and *CORT* genes and correlated with their gene expression levels, were associated with asthma exacerbations despite ICS treatment.
					Replication, (1) 72 (2) 168		Replication, (1) EUR, (2) LAT		
Wang et al^24^	2019	EWAS (27K array)	ICS	Drug response	152	5–12 y	EUR	Whole blood	Hypo- and hypermethylation at *BOLA2* and *OTX2*, respectively, were associated with FEV_1_ improvement after 8 wk of ICS treatment.
Kere et al^14^	2020	EWAS (450K array)	ICS	Effects on DNAm	Discovery, 215	8–16 y	EUR	Whole blood	24 CpGs were associated with ICS treatment, but none was replicated.
					Replication, (1) 173, (2) 96				
Nwanaji-Enwerem et al^20^	2022	EWAS (450K array)	SCS	Drug response	20	5–18 y	EUR 20%, AFR 70%, MIX 10%	Nasal brushings	Epigenetic age acceleration was associated with OCS response in a sex-specific manner. Female subjects experiencing good response had lower epigenetic age acceleration than male subjects without an adequate response.
Slob et al^29^	2022	EWAS (EPIC array)	ICS	meQTL of ICS-induced CpGs	Discovery, 43	Discovery, Adults	Discovery, Recruited in Netherlands	Bronchial biopsy	76 meQTL associated with CpGs affected by ICS treatment were identified in adults with COPD. None was associated with response to ICS in pediatric asthma.
					Evaluation, (1) 1515, (2) 1702	Evaluation. Children	Evaluation EUR, LAT 59%, AA 33%, EAS 8%		
Cardenas et al^21^	2019	EWAS (EPIC array)	Albuterol	Drug response	454	12–15 y	EUR 67.1%, AFR 16.1%, LAT 4.2%, EAS 3.1%, MIX 9.3%	Nasal brushings	130 CpGs were genome-wide significantly associated with BDR. These were not associated with other asthma traits.
Martin-Almeida et al^32^	2023	EWAS (EPIC array)	Albuterol	Drug response	121	6–17 y	EUR 79%, LAT 8%, AFR 3%, EAS 2%, MIX 8%	Whole blood	4 DMRs (*C5orf63, PPFIBP2, RAMP1, LY6G5C*) were significantly associated with BDR in pediatric asthma. 3 CpGs (*ADD3-AS1, PARP2, FAM69B*) were suggestively associated with BDR (FDR < 0.1). Epigenetic signals were enriched in IL-5, IL-2, FcεRI, and aging pathways.
Perez-Garcia et al^30^	2023	EWAS (EPIC array)	Albuterol	Drug response	Discovery, 221	8–21 y	Discovery, AA	Whole blood	A CpG on *FGL2* was associated with BDR in AA and regulated by 7 meQTL. A CpG on *DNASE2* was associated with BDR in AA and LAT and acts as eQTM of *RNASEH2A*. 5 DMRs regulated by genetic variation (*CEBPD, ZNF205, CDKN1C, FGL2, GUSB*) were associated with BDR in AA.
					Replication, 193		Replication, LAT		
Perez-Garcia et al^31^	2023	EWAS (EPIC array)	Albuterol	Effects on DNAm (*in vitro*)	Discovery, 97	Discovery, 8–21 y	Discovery, LAT	Discovery, NEC validation, NEC,	22 CpGs and 9 DMRs were associated with albuterol treatment. Effects on *CREB3L1, MYLK4*, and *KSR1* were replicated and associated with genetic variation. Hypomethylation at *KSR1* and *FLNC* was associated with increased gene expression. Hypomethylation at *CREB3L1* was validated in BEC and associated with BDR.
					Validation, 10 (paired samples)	Validation, Adults	Validation, EUR 60%, LAT 20%, EAS 20%	BEC	
Wang et al^43^	2018	Candidate-gene	SIT	Effects on DNAm	37	6–18 y	Recruited in Taiwan	PBMCs	Mite-SIT increased DNAm at *IL4* promoter, while no effects were observed in *IL2, IL13, CCL17, IFNG*, and *RUNX3*.
Wang et al^44^	2020	EWAS (450K array)	SIT	Effects on DNAm	25 (analyzed as DNA pools)	6–18 y	Recruited in Taiwan	PBMCs	Mite-SIT induced DNAm changes in 108 CpGs at nominal level involved in antigen presentation, apoptosis, and extracellular matrix. Targeted DNAm and gene expression analyses validated hypomethylation and increased expression of *BCL6*, and hypermethylation at *HSPG2* and *HSP90AA1* with decreased *HSPG2* expression.
Rakkar et al^48^	2024	EWAS (EPIC array)	Mepolizumab	Effects of DNAm and drug response	27 (longitudinal)	Adults	Recruited in United Kingdom	Nasal brushings	53 CpGs were differentially methylated after 3 mo mepolizumab treatment, of which 42 were associated with gene expression. Baseline DNAm was not associated with drug response, and longitudinal DNAm changes were not evident on stratification of those with and without response to mepolizumab.

In case of studies evaluating multiple traits, only descriptives corresponding to therapy-related analyses are reported. Proportion of different ethnic groups is estimated for individuals included in therapy-related analyses. In case it could not be estimated based on available data, proportions are indicated as reported in original document for whole population. *27K array*, Infinium HumanMethylation 27K BeadChip; *450K array*, Infinium HumanMethylation 450K BeadChip; *AA*, African American; *AFR*, African; *BEC*, bronchial epithelial cells; *COPD*, chronic obstructive pulmonary disease; *EAS*, East Asian; *EPIC array*, Infinium HumanMethylationEPICv1 BeadChip; *EUR*, European descendants; *FEV_1_*, forced expiratory volume in 1 second; *GC*, glucocorticosteroid; *LAT*, Latino; *MIX*, mixed populations; *NCS*, nasal steroids; *PBMCs*, peripheral blood mononuclear cells; *SCS*, systemic corticosteroids.

**TABLE II.** Summary of microbiome studies of asthma therapies published until April 2024

Study	Year	Approach	Therapy	Outcome	Sample size	Age group	Ancestry	Biological sample	Main findings

Goleva et al^63^	2013	16S rRNA (V1-V2)	OCS	Drug response	39	Adults	EUR 72%, AFR 10%, Other 18%	Lung (BAL)	OCS response (based on FEV_1_ improvement after 7 d of treatment) was not associated with microbiome diversity and bacteria load. Bacterial genera with high (*Haemophilus*) and low (*Fusobacterium*) endotoxic activity might be positively and inversely associated with steroid resistance, respectively.
Huang et al^54^	2015	16S rRNA (PhyloChip platform)	ICS	Effects on microbiome	71 (2 combined cohorts)	Adults	Severe asthma, EUR 80% Other 20%	Lung (EB)	Severe asthma patients treated with high-dose ICS showed ↑Actinobacteria and ↑ Gammaproteobacteria (*Klebsiella*), and ↓Proteobacteria than mild-moderate asthma patients treated with low-dose ICS. Actinobacteria and α-diversity were positively associated with *FKBP5* expression, a marker of steroid response.
							Mild asthma, NA		
Denner et al^55^	2016	16S rRNA (V4)	ICS, OCS	Effects on microbiome	39	Adults	EUR 54%, AA 44%, Other 2%	Lung (EB, BAL)	ICS-treated patients had ↓*Prevotella* and ↓ *Veillonella* than steroid-naive patients. Add-on OCS therapy expands alterations to *Rickettsia, Pseudomonas, Lactobacillus*, and *Streptococcus*.
Durack et al^56^	2017	16S rRNA (V4)	ICS	Effects on microbiome and drug response	25 cross-sectional, 8 longitudinal	Adults	EUR 60%, Other 40%	Lung (EB)	Subjects with good response to ICS (≥2-fold increase in PC_20_Mch after ICS treatment) showed ↑Streptococcaceae, ↑Fusobacteriaceae, ↑Sphingomonadaceae, ↓Microbacteriaceae, and ↓Pasteurellaceae (*Haemophilus*) compared to those with a poor response. Six weeks of ICS treatment in those having good response did not alter bacterial diversity but rather composition (↑*Neisseria*, ↓*Moraxella*, ↓Microbacteriaceae, ↓*Fusobacterium*).
Goleva et al^76^	2017	16S rRNA (V1-V2)	OCS	Drug response (*in vitro*)	NA	NA	Recruited in Colorado	Lung (BEC)	Opportunistic pathogens of *Streptococcus mitis group* reduce cellular response to dexamethasone via p38 MAPK in bronchial epithelia compared to commensal *S oralis.*
Turturice et al^58^	2017	Shotgun metagenomics	ICS	Effects on microbiome	13 longitudinal	Adults	Recruited in Australia	Lung (BAL)	Six-week ICS treatment was associated with ↑lung bacterial diversity and ↓*Streptococcus pneumoniae*, ↓*Neisseria meningitidis*, ↓*Enterococcus faecium*, and ↓*E faecalis.* These bacteria were associated with IL-2, TNF-α, and MIP-1β levels before ICS treatment.
Sharma et al^60^	2019	16S rRNA (V4)	ICS, OCS	Effects on microbiome	39	Adults	EUR 56%, AFR 41%, Other 3%	Lung (EB, BAL)	ICS and OCS treatments were associated with changes in asthma endotype-related bacteria in EB (↓*Atopobium*, ↓*Stenotrophomonas*, ↓*Actinomyces*). ICS treatment was also associated with ↑*Acinetobacter*, ↑*Rickettsiales*, ↑Rhodobacteraceae, and ↓Lachnospiracease in EB, and ↑*Herbaspirillum*, ↑Rhodobacteraceae, ↑*Pseudomonas*, and ↑*Alteromonas* in BAL. Affected bacterial metabolic pathways included MAPK signaling, bile acid metabolism, ABC transporters, and ubiquitin-proteasome system.
Zhou et al^68^	2019	16S rRNA (V1-V3)	ICS	Drug response	214	5–11 y	EUR 57%, Other 43%	Upper airways (NS)	Bacteria load and α-diversity were associated with reduced risk for exacerbations despite low-dose ICS treatment. Children with nasal microbiome dominated by *Corynebacterium* and *Dolosigranulum* had reduced risk of loss of asthma control episodes than those with a microbiome dominated by *Staphylococcus, Streptococcus*, or *Moraxella*.
Durack et al^57^	2020	16S rRNA (V4)	ICS	Effects on microbiome and drug response	31	Adults	EUR 56%, Other 44%	Oral wash and sputum	Sputum bacterial phylogenetic diversity was reduced after 6 wk of ICS treatment, with higher deviations from baseline in those with a poor response to ICS than those with a good response (ICS responsiveness defined as increase in PC_20_Mch after ICS treatment). Bacterial communities in terms of β-diversity were distinct between those with good and poor response.
Huang et al^52^	2020	16S rRNA (V3-V4)	ICS	Effects on microbiome	56	Adults	Recruited in China	Sputum	ICS treatment reduces sputum abundances of potential pathogenic bacteria enriched in asthma patients compared to controls (*Streptococcus, Gemella, Neisseria*). ICS did not reverse the reduction of fungal-bacterial interactions observed in asthma patients compared to healthy subjects.
Martin et al^59^	2020	16S rRNA (V3-V4)	ICS	Effects on microbiome	55	Adults	EUR 96%, AFR 4%	Sputum	Subjects treated with low-dose ICS showed ↑*Streptococcus* and ↑*Prevotella* abundances; those treated with high-dose ICS showed ↑*Dysgonomonas* and ↑*Parvimonas*. At high doses, fluticasone was associated with ↑*Haemophilus parainfluenzae* compared to budesonide. No effects were observed for bacteria load and diversity.
Huang et al^53^	2022	16S rRNA (V3-V4)	ICS	Effects on microbiome	14 longitudinal	Adults	Recruited in China	Sputum	Nine-month treatment with ICS showed no effects on sputum microbiome diversity and composition. ICS treatment reduced complexity of bacterial and fungal interactions.
Perez-Garcia et al^70^	2023	16S rRNA (V3-V4)	ICS	Drug response	250	8–85 y	EUR	Discovery, Upper airways (saliva, NS, PS)	Asthma exacerbations were associated with lower bacterial diversity and abundance of 18 bacterial genera (↓*Bifidobacterium*, ↓*Selenomonas*, ↓*Porphyromonas*) in the upper airways. The salivary microbiome improves clinical and genetic classification models of asthma exacerbations.
Perez-Garcia et al^77^	2023	16S rRNA (V3-V4)	ICS	mbGWAS of response to ICS -related bacteria	Discovery, 257	Discovery, 8–85 y	Discovery, EUR	Discovery, Upper airways (saliva, NS, PS)	Genes associated with exacerbation- related bacteria were enriched in asthma comorbidities (reflux esophagitis, obesity, tobacco use disorder) and were likely regulated by trichostatin A and NF-κB, glucocorticosteroid receptor, and C/EBP transcription factors.
					Replication, (1) 158,(2) 114	Replication, 8–21 y	Replication, (1) LAT, (2) AA	Replication, Saliva	
Slater et al^82^	2014	16S rRNA (V4-V7 pyrosequencing)	Azithromycin	Effects on microbiome	5 longitudinal	Adults	Recruited in UK	Lung (BAL)	Azithromycin increased *Anaerococcus* presence in lung microbiome and decreased potential pathogenic bacteria (*Pseudomonas, Haemophilus, Staphylococcus*).
Lopes Dos Santos Santiago et al^86^	2017	16S rRNA (V1-V2)	Azithromycin	Effects on microbiome	13 longitudinal	Adults	EUR	Upper airways (PS)	Long-term azithromycin treatment was associated with ↑*Streptococcus salivarius* and ↓*Leptotrichia wadei*. Microbiomes were recovered 1 mo after treatment cessation.
Wei et al^83^	2018	16S rRNA (V4)	Azithromycin	Effects on microbiome	59 short term, 49 long term	1–4 y	EUR 98%, Other 2%	Feces	Azithromycin induces nondurable perturbation of gut microbiome (↓α-diversity and ↓*Bifidobacterium*).
Park et al^84^	2020	16S rRNA (V3-V4)	Azithromycin	Effects on microbiome (murine model)	NA	NA	NA	Lung (BAL) and feces	Azithromycin reduces airway inflammation through increasing SCFA producers (Clostridiales, such as Ruminococcaceae and Lachnospiraceae). Acetate was shown to diminish lung inflammatory markers. These effects were only observed during gut microbiome maturation.
Thorsen et al^87^	2021	16S rRNA (V4)	Azithromycin	Drug response	68 longitudinal	1–3 y	EUR 97%, Other 3%	Upper airways (PA)	Azithromycin response is increased by higher baseline bacterial richness, increased abundances of *Veillonella, Leuconostoc*, and *Vibrio* OTUs, and depletion of some *Neisseria* OTUs.
Diver et al^89^	2022	16S rRNA (V4)	Mepolizumab	Effects on microbiome	13 longitudinal	Adults	EUR	Sputum	Twelve weeks' treatment with mepolizumab did not alter sputum bacteria load, diversity, and composition, with no effects on pathogenic bacteria abundances and Proteobacteria:Firmicutes ratio.

In case of studies evaluating multiple traits, only descriptives corresponding to therapy-related analyses are indicated. Proportion of different ethnic groups is estimated for individuals included in therapy-related analyses. In case it could not be estimated based on available data, proportions are indicated as reported in original documents for whole population. *AA*, African American; *AFR*, African; *BAL*, bronchoalveolar lavage; *BEC*, bronchial epithelial cells; *EB*, bronchial epithelial brushing; *EUR*, European descendants; *FEV_1_*, forced expiratory volume in 1 second; *LAT*, Latino; *NA*, not applicable; *NS*, nasal swab; *OTU*, operational taxonomic unit; *PA*, hypopharyngeal aspirate; *PC_20_Mch*, provocative dose of methacholine that results in 20% decrease in *FEV_1_*; PS, pharyngeal swab; *rRNA*, ribosomal RNA.

## Box 1. Description of systematic literature search methodology

### Search strategy

A systematic literature review was carried out using the PubMed database with different combinations of keywords comprising the terms “microbiome,” “epigenetics,” and “asthma treatments.” A 3-stage screening was performed to select eligible studies according to adequacy of title, abstract, and full text.

### Keywords

- Asthma.
- Methylation, epigenetics, epigenomics, EWAS, microbiome, microbiota.
- Albuterol, salbutamol, bronchodilator, corticosteroids, glucocorticosteroids, steroids, montelukast, antimuscarinic, antihistamine, azithromycin, immunotherapy, omalizumab, mepolizumab, benralizumab, dupilumab.

### Inclusion criteria

- Publication date until April 30, 2024.
- Microbiome and epigenetic studies examining associations with asthma therapies or treatment response.
- Manuscripts written in English.

### Exclusion criteria

- Not including asthma patients or not aimed at investigating asthma in humans.
- Addressing therapies not indicated for asthma treatment according to international guidelines.
- Not based on high-throughput microarray or DNA sequencing technologies.
- Focused exclusively on the mycobiome or virome.
- Reviews, editorials, opinion articles, or case studies.

## Box 2. Brief description of some therapeutic approaches targeting methylation markers or microbiome

### Methylation markers

#### CRISPR/dCas9 systems.

A CRISPR system based on a deactivated Cas9 without nuclease activity. These systems recognize a target DNA sequence allowing the guide of specific enzymes to exert an action. In the epigenetic field, enzymes with epigenetic actions can be directed to modulate epigenetic markers in specific loci (eg, methyltransferases can be used to modify DNAm patterns in a specific sequence).

#### DNAm inhibitors.

Molecules that directly or indirectly inhibit DNAm—for example, through irreversible binding to methyltransferases. These are often classified into nucleoside (eg, 5-azacytidine) and nonnucleoside analogs.

### Microbiome

#### Bacterial lysates.

Immunomodulating agents consisting of bacterial pathogens lysates that stimulate both the innate and adaptive immunity when administered, resulting in increased host immune defense.

#### Bacterial monoclonal therapies.

Immunoglobulins with high specificity for a bacterial antigen or epitope.

#### Bacteriophages.

Viruses that target specific bacterial strains or species. These are the dominant components of the human virome that could be therapeutically administered to regulate the abundance of specific bacteria selectively.

#### Fecal microbiota transplantation.

Administration of a solution of fecal or gut matter from a donor into a recipient’s intestinal tract to induce microbiome changes with therapeutic effects.

#### Postbiotics.

Inanimate microorganisms and/or cell components that have beneficial effects on the host. This term may also include microbial-derived metabolites and molecules, such as vitamins, amino acids, or SCFAs.

#### Prebiotics.

Nondigestible food components that influence microbial communities’ composition by selectively stimulating the growth or activity of specific microorganisms.

#### Probiotics.

Living microorganisms with potential therapeutic benefits when consumed or applied to the body.

## Abbreviations used

BDR: Bronchodilator drug response
C/EBP: CCAAT/enhancer-binding proteins
CCL: Chemokine C-C motif ligand
DMR: Differentially methylated region
DNAm: DNA methylation
eQTM: Expression quantitative trait methylation
EWAS: Epigenome-wide association study
ICS: Inhaled corticosteroids
MAPK: Mitogen-activated protein kinase
mbGWAS: Microbiome genome-wide association study
mbQTL: Microbiome quantitative trait loci
meQTL: Methylation quantitative trait loci
miRNA: MicroRNA
NF-κB: Nuclear factor kappa–light-chain enhancer of activated B cells
OCS: Oral corticosteroid
SCFA: Short-chain fatty acid
SIT: Specific allergen immunotherapy
T2: Type 2
