# Supplementary material for: Precision medicine for asthma treatment: Unlocking the potential of the epigenome and microbiome
Source: J Allergy Clin Immunol. Author manuscript; Available in PMC 2026 Feb 1. (PMC12002393; doi:10.1016/j.jaci.2024.06.010)
Supplement: Supplemental text and Fig E1 [file NIHMS2067497-supplement-Supplemental_text_and_Fig_E1.pdf]

Online Repository

Precision Medicine for Asthma Treatment: Unlocking the Potential of the Epigenome and Microbiome

Javier Perez-Garcia, PhD*, Andres Cardenas, PhD, Fabian Lorenzo-Diaz, PhD†, Maria Pino-Yanes, PhD†

†*Equal contribution as senior authors.*

**Corresponding author:*

Javier Perez-Garcia, PhD
Genomics and Health Group, Department of Biochemistry, Microbiology, Cell Biology, and Genetics, Universidad de La Laguna (ULL). Apartado 456, La Laguna, 38200 Santa Cruz de Tenerife, Spain.
Tel: +34 922316502–8350. Email: jpegarci@ull.edu.es

SUPPLEMENTARY DATA

Potential pathways suggested to underly albuterol effects through DNA methylation changes

The only study examining albuterol-induced DNA methylation (DNAm) in airway epithelia reported novel evidence of genome-wide DNAm changes induced *in vitro* by albuterol in nasal epithelial cells (E1). Encoded proteins by affected genes were hypothesized to potentially underly the biological effects of albuterol, mainly participating in the β_2-adrenergic receptor (ADRB2) pathway (E2) (**Figure 5**).

Three epigenetic loci affected by albuterol with evidence of replication were annotated to the cAMP responsive element binding protein 3 like 1 (*CREB3L1*), myosin light chain kinase family member 4 (*MYLK4*), and kinase suppressor of ras 1 (*KSR1*) genes (E1). These three genes are moderate to highly expressed in lungs and smooth muscle tissues (E3). The effect on the CpG on *CREB3L1* was observed in nasal but also bronchial epithelia. *CREB3L1* encodes one member of the cAMP response element-binding (CREB) transcription factor family. These proteins regulate the expression of genes containing a cAMP-responsive element (CRE) in response to cAMP, being therefore mechanistically regulated by ADRB2 (E2,E4). *CREB3L1* is highly expressed in human mucus secretory cells (E5), being implicated in stress response, cell secretory capacity and migration, extracellular matrix production, and host defense (E6E9).

The hitherto unknown relationship of *CREB3L1* with bronchodilators is supported by the influence of the family-related CREB1 protein in asthma inflammation, airway smooth muscle contraction, tolerance to bronchodilators, and the agonism of β_2-bronchodilators and glucocorticosteroids (E10E12). Furthermore, CREB1 modulates the immune system by regulating CRE-containing genes (*e.g., IL2* and *TNF*), immune cells proliferation, the balance among Th1, Th2, and Th17 responses, and the NF-κB inflammatory response (E4). Additionally, this study reported an enrichment of proteins (including CREB3L1) interacting with the histone acetyltransferase E1A binding protein p300 (EP300). EP300 is a coactivator closely related to the regulatory effect of CREB on the NF-κB pathway, with the activation of this pathway being determined by the balance between CREB (inhibition) and EP300 (enhancement) (E4).

In addition, the cAMP-dependent protein kinase A (PKA) is implicated in multiple processes in airway cells, such as mucociliary clearance, inhibition of inflammatory cells, and bronchodilation (E13). In airway smooth muscle, the PKA-mediated reduction in Ca^{2+} levels and sensitivity results in the inhibition of MYLKs and the subsequent rapid-onset bronchodilator effect of β_2-agonists (E2). This study reported that

albuterol affects DNAm at a CpG annotated to *MYLK4* as the nearest protein-coding gene (E1). *MYLK4* encodes a protein from the MYLK family, constituted by different kinases (i.e., from MYLK1 to MYLK4) that participate in dynamic changes in the cytoskeleton involved in cell migration, invasion, and proliferation (E14,E15). Although the function of *MYLK4* has been scarcely studied (E16,E17), its influence on asthma might be supported by the fact that another *MYLK* gene, *MYLK1*, is involved in asthma onset and exacerbations, smooth muscle contraction, and airway inflammation through genetic and epigenetic variations (E18E20).

The potential effect of albuterol on actin cytoskeleton components and cell proliferation via epigenetics was also described in enrichment analyses and supported genome-wide association findings (E1). Noteworthily, the top hit epigenetic association in this study was mapped as an expression quantitative trait methylation (eQTM) of the filamin C (*FLNC*) gene. FLNC participates in the anchoring of the actin cytoskeleton to membrane proteins and is expressed in cardiac, smooth, and striated muscle cells, being primarily localized in the muscle Z-discs (E21). Alterations in FLNC activity have been mainly related to the development of cardiomyopathy and muscle weakness (E22), but their role in albuterol-mediated bronchodilation has not been described. Nonetheless, the fact that FLNC binds to proteins involved in smooth muscle tone (*e.g.,* β-arrestin-2 and the α_1-adrenergic receptor) suggest a potential interaction with albuterol (E22). Specifically, the β-arrestin-2 blocks the bound of G proteins to ADRB2, and it is involved in asthma development and side effects of β_2-agonists (*i.e.,* reduced bronchodilation and pro-inflammatory effect) (E2).

Additionally, genome-wide significant results also included the transforming growth factor-β (TGF-β) induced (*TGFBI*) gene. TGFBI is an extracellular protein involved in cell adhesion, with a relevant role in regulating lung function (E23). Indeed, TGFBI has been associated with lung development, lung fibrosis, and pulmonary diseases such as bronchopulmonary dysplasia (E23,E24). This protein is activated by the TGF-β1, a growth factor and cytokine elevated in asthma patients involved in airway remodeling, inflammation, and hyperresponsiveness (E25). TGF-β1 regulates airway smooth muscle cell proliferation and contraction, with the latter effect being mediated via SMAD3 (E25). SMAD3 is an intracellular signal transducer closely related to asthma. Specifically, genetic variation in *SMAD3* was associated with asthma susceptibility in the first GWAS of asthma (E26), while DNAm of a DMR in the promoter region of this gene has been associated with asthma risk and IL-1β levels in children with

maternal asthma (E27). Enrichment analyses of the study by Perez-Garcia et al. also showed a potential implication of albuterol on the SMAD3 pathway (E1), which is supported by the fact that *SMAD3* is upregulated by albuterol, with potential consequences on muscle cell growth (E28).

The study by Perez-Garcia et al. also supported that the mitogen-activated protein kinase (MAPK) pathway could be affected by albuterol through DNAm changes (E1). Indeed, a genome-wide significant epigenetic locus was found to be associated with the expression of *KSR1*, which encodes a scaffold protein that couples Ca^{2+} and calmodulin to modulate the activation of MAPK signaling (E29). KSR1 has also been implicated in host defense against respiratory infections through nitric oxide release (E30), and DNAm on *KSR1* has been inversely associated with immunoglobulin E (IgE) levels during childhood (E31). Additionally, enrichment analyses showed that suggestive DNAm associations with albuterol treatment were annotated to genes encoding proteins that likely interact with MAPK1, MAPK3, and MAPK9. The MAPK pathway is implicated in asthma through the production of cytokines and pro-inflammatory mediators, immune cell activation and migration, and eosinophil degranulation (E32). Furthermore, the self-perpetuated activation of MAPK by repeated stimulations (*e.g.,* viral infections or allergen exposure) in bronchial epithelial cells is a potential mechanism leading to airway inflammation and asthma onset (E32). Moreover, β_2-agonists activate the MAPK pathway via β-arrestins, which mediates the IgE production and airway inflammation induced by these drugs (E2,E33).

Finally, regional DNAm, methylation QTL (meQTL), and *in silico* expression QTL (eQTL) analyses further supported the potential effect of albuterol on *CREB3L1*, *MYLK4*, and *KSR1* loci (E1). Differentially methylated regions associated with albuterol treatment were identified for these three epigenetic loci, being the effect on the top CpGs regulated by three SNPs that act as eQTLs in airway tissue. Specifically, two of these SNPs regulated genes where they are located (*i.e., LINC01600* and *KSR1*). However, these analyses also revealed the potential effect of albuterol on other candidate genes and mechanisms. On one hand, the study by Perez-Garcia et al. (E1) reported a DMR and a genome-wide CpG on the period circadian regulator 2 (*PER2*), a member of the Period gene family involved in circadian rhythm regulation. Dysregulation of circadian rhythm genes (*e.g., PER2* and *CLOCK*) has been associated with nocturnal asthma (E34). Furthermore, β_2-agonists, which are effective in treating nocturnal asthma (E35), regulate these genes via CREB (E36). Additionally, an eQTL of *CLOCK* and *PER2* was associated with bronchodilator drug response in a genome-wide association study of children with asthma (E37). On the

other hand, this study identified a meQTL (annotated to *CREB3L1*) that regulates the expression of the autophagy-related 13 (*ATG13*) gene in lungs. *ATG13* encodes a factor involved in autophagy and the mammalian target of the rapamycin pathway, two processes associated with increased risk for asthma onset and exacerbations (E38,E39). Enrichment analyses also suggested a potential implication of albuterol on autophagy regulation via epigenetics, which could be supported by the fact that ADRB2 regulates this mechanism in other tissues (E40).

SUPPLEMENTARY REFERENCES

E1. Perez-Garcia J, Pino-Yanes M, Plender EG, Everman JL, Eng C, Jackson ND, et al. Epigenomic response to albuterol treatment in asthma-relevant airway epithelial cells. Clin Epigenetics. 2023;15(1):156.

E2. Wendell SG, Fan H, Zhang C. G Protein–Coupled Receptors in Asthma Therapy: Pharmacology and Drug Action. Pharmacol Rev. 2020;72(1):1–49.

E3. Papatheodorou I, Moreno P, Manning J, Fuentes AMP, George N, Fexova S, et al. Expression Atlas update: from tissues to single cells. Nucleic Acids Res. 2020;48(D1):D77–83.

E4. Wen AY, Sakamoto KM, Miller LS. The Role of the Transcription Factor CREB in Immune Function. J Immunol. 2010;185(11):6413–9.

E5. Goldfarbmuren KC, Jackson ND, Sajuthi SP, Dyjack N, Li KS, Rios CL, et al. Dissecting the cellular specificity of smoking effects and reconstructing lineages in the human airway epithelium. Nat Commun. 2020;11(1):2485.

E6. Kamikawa Y, Saito A, Matsuhisa K, Kaneko M, Asada R, Horikoshi Y, et al. OASIS/CREB3L1 is a factor that responds to nuclear envelope stress. Cell Death Discov. 2021;7(1):152.

E7. Fox RM, Hanlon CD, Andrew DJ. The CrebA/Creb3-like transcription factors are major and direct regulators of secretory capacity. J Cell Biol. 2010;191(3):479–92.

E8. Vellanki RN, Zhang L, Volchuk A. OASIS/CREB3L1 is induced by endoplasmic reticulum stress in human glioma cell lines and contributes to the unfolded protein response, extracellular matrix production and cell migration. PLoS One. 2013;8(1):e54060.

E9. Denard B, Seemann J, Chen Q, Gay A, Huang H, Chen Y, et al. The membrane-bound transcription factor CREB3L1 is activated in response to virus infection to inhibit proliferation of virus-infected cells. Cell Host Microbe. 2011;10(1):65–74.

E10. Chiappara G, Chanez P, Bruno A, Pace E, Pompeo F, Bousquet J, et al. Variable p-CREB expression depicts different asthma phenotypes. Allergy. 2007;62(7):787–94.

E11. Barnes PJ. Scientific rationale for inhaled combination therapy with long-acting β2-agonists and corticosteroids. Eur Respir J. 2002;19(1):182–91.

E12. Yadav SK, Sharma P, Shah SD, Panettieri RA, Kambayashi T, Penn RB, et al. Autocrine regulation of airway smooth muscle contraction by diacylglycerol kinase. J Cell Physiol. 2022;237(1):603–16.

E13. Billington CK, Ojo OO, Penn RB, Ito S. cAMP Regulation of Airway Smooth Muscle Function. Pulm Pharmacol Ther. 2013;26(1):112–20.

E14. Li HS, Lin Q, Wu J, Jiang ZH, Zhao JB, Pan J, et al. Myosin regulatory light chain phosphorylation is associated with leiomyosarcoma development. Biomed Pharmacother. 2017;92:810–8.

E15. Kamm KE, Stull JT. Signaling to Myosin Regulatory Light Chain in Sarcomeres. J Biol Chem. 2011;286(12):9941–7.

E16. Lee RDW, Song MY, Lee JK. Large-scale profiling and identification of potential regulatory mechanisms for allelic gene expression in colorectal cancer cells. Gene. 2013;512(1):16–22.

E17. Herrer I, Roselló-Lletí E, Rivera M, Molina-Navarro MM, Tarazón E, Ortega A, et al. RNA-sequencing analysis reveals new alterations in cardiomyocyte cytoskeletal genes in patients with heart failure. Lab Invest. 2014;94(6):645–53.

E18. Acosta-Herrera M, Pino-Yanes M, Ma SF, Barreto-Luis A, Corrales A, Cumplido J, et al. Fine mapping of the myosin light chain kinase (MYLK) gene replicates the association with asthma in populations of Spanish descent. J Allergy Clin Immunol. 2015;136(4):1116–1118.e9.

E19. Gao L, Grant A V., Rafaels N, Stockton-Porter M, Watkins T, Gao P, et al. Polymorphisms in the myosin light chain kinase gene that confer risk of severe sepsis are associated with a lower risk of asthma. J Allergy Clin Immunol. 2007;119(5):1111–8.

E20. Sun X, Sun BL, Sammani S, Bermudez T, Dudek SM, Camp SM, et al. Genetic and epigenetic regulation of the non-muscle myosin light chain kinase isoform by lung inflammatory factors and mechanical stress. Clin Sci (Lond). 2021;135(7):963–77.

E21. Nakamura F, Stossel TP, Hartwig JH. The filamins: Organizers of cell structure and function. Cell Adh Migr. 2011;5(2):160–9.

E22. Mao Z, Nakamura F. Structure and Function of Filamin C in the Muscle Z-Disc. Int J Mol Sci. 2020;21(8):2696.

E23. Yang K, Huang N, Sun J, Dai W, Chen M, Zeng J. Transforming growth factor-β induced protein regulates pulmonary fibrosis via the G-protein signaling modulator 2 /Snail axis. Peptides. 2022;155:170842.

E24. Ahlfeld SK, Wang J, Gao Y, Snider P, Conway SJ. Initial Suppression of Transforming Growth Factor-β Signaling and Loss of TGFBI Causes Early Alveolar Structural Defects Resulting in Bronchopulmonary Dysplasia. Am J Pathol. 2016;186(4):777–93.

E25. Ojiaku CA, Yoo EJ, Panettieri RA. Transforming growth factor β1 function in airway remodeling and hyperresponsiveness: The missing link? Am J Respir Cell Mol Biol. 2017;56(4):432–42.

E26. Moffatt MF, Gut IG, Demenais F, Strachan DP, Bouzigon E, Heath S, et al. A Large-Scale, Consortium-Based Genomewide Association Study of Asthma. N Engl J Med. 2010;363(13):1211–21.

E27. DeVries A, Wlasiuk G, Miller SJ, Bosco A, Stern DA, Lohman IC, et al. Epigenome-wide Analysis Links SMAD3 Methylation at Birth to Asthma in Children of Asthmatic Mothers. J Allergy Clin Immunol. 2017;140(2):534–42.

E28. Hostrup M, Reitelseder S, Jessen S, Kalsen A, Nyberg M, Egelund J, et al. Beta2-adrenoceptor agonist salbutamol increases protein turnover rates and alters signalling in skeletal muscle after resistance exercise in young men. J Physiol. 2018;596(17):4121–39.

E29. Parvathaneni S, Li Z, Sacks DB. Calmodulin influences MAPK signaling by binding KSR1. J Biol Chem. 2021;296:100577.

E30. Zhang Y, Li X, Carpinteiro A, Goettel JA, Soddemann M, Gulbins E. Kinase suppressor of Ras-1 protects against pulmonary Pseudomonas aeruginosa infections. Nat Med. 2011;17(3):341–6.

E31. Peng C, Cardenas A, Rifas-Shiman SL, Hivert MF, Gold DR, Platts-Mills TA, et al. Epigenome-wide association study of total serum immunoglobulin E in children: a life course approach. Clin Epigenetics. 2018;10:55.

E32. Alam R, Gorska MM. MAPK Signaling and ERK1/2 bistability in Asthma. Clin Exp Allergy. 2011;41(2):149–59.

E33. Pongratz G, McAlees JW, Conrad DH, Erbe RS, Haas KM, Sanders VM. The level of IgE produced by a B cell is regulated by norepinephrine in a p38 MAPK- and CD23-dependent manner. J Immunol. 2006;177(5):2926–38.

E34. Chen HC, Chen YC, Wang TN, Fang WF, Chang YC, Chen YM, et al. Disrupted Expression of Circadian Clock Genes in Patients with Bronchial Asthma. J Asthma Allergy. 2021;14:371–80.

E35. Greenberg H, Cohen RI. Nocturnal asthma. Curr Opin Pulm Med. 2012;18(1):57–62.

E36. Travnickova-Bendova Z, Cermakian N, Reppert SM, Sassone-Corsi P. Bimodal regulation of mPeriod promoters by CREB-dependent signaling and CLOCK/BMAL1 activity. Proc Natl Acad Sci U S A. 2002;99(11):7728–33.

E37. Duan QL, Lasky-Su J, Himes BE, Qiu W, Litonjua AA, Damask A, et al. A genome-wide association study of bronchodilator response in asthmatics. Pharmacogenomics J. 2014;14(1):41–7.

E38. Ban GY, Pham DL, Trinh THK, Lee SI, Suh DH, Yang EM, et al. Autophagy mechanisms in sputum and peripheral blood cells of patients with severe asthma: a new therapeutic target. Clin Exp Allergy. 2016;46(1):48–59.

E39. Zhang Y, Jing Y, Qiao J, Luan B, Wang X, Wang L, et al. Activation of the mTOR signaling pathway is required for asthma onset. Sci Rep. 2017;7(1):4532.

E40. Lizaso A, Tan KT, Lee YH. β-adrenergic receptor-stimulated lipolysis requires the RAB7-mediated autolysosomal lipid degradation. Autophagy. 2013;9(8):1228–43.

SUPPLEMENTARY FIGURES

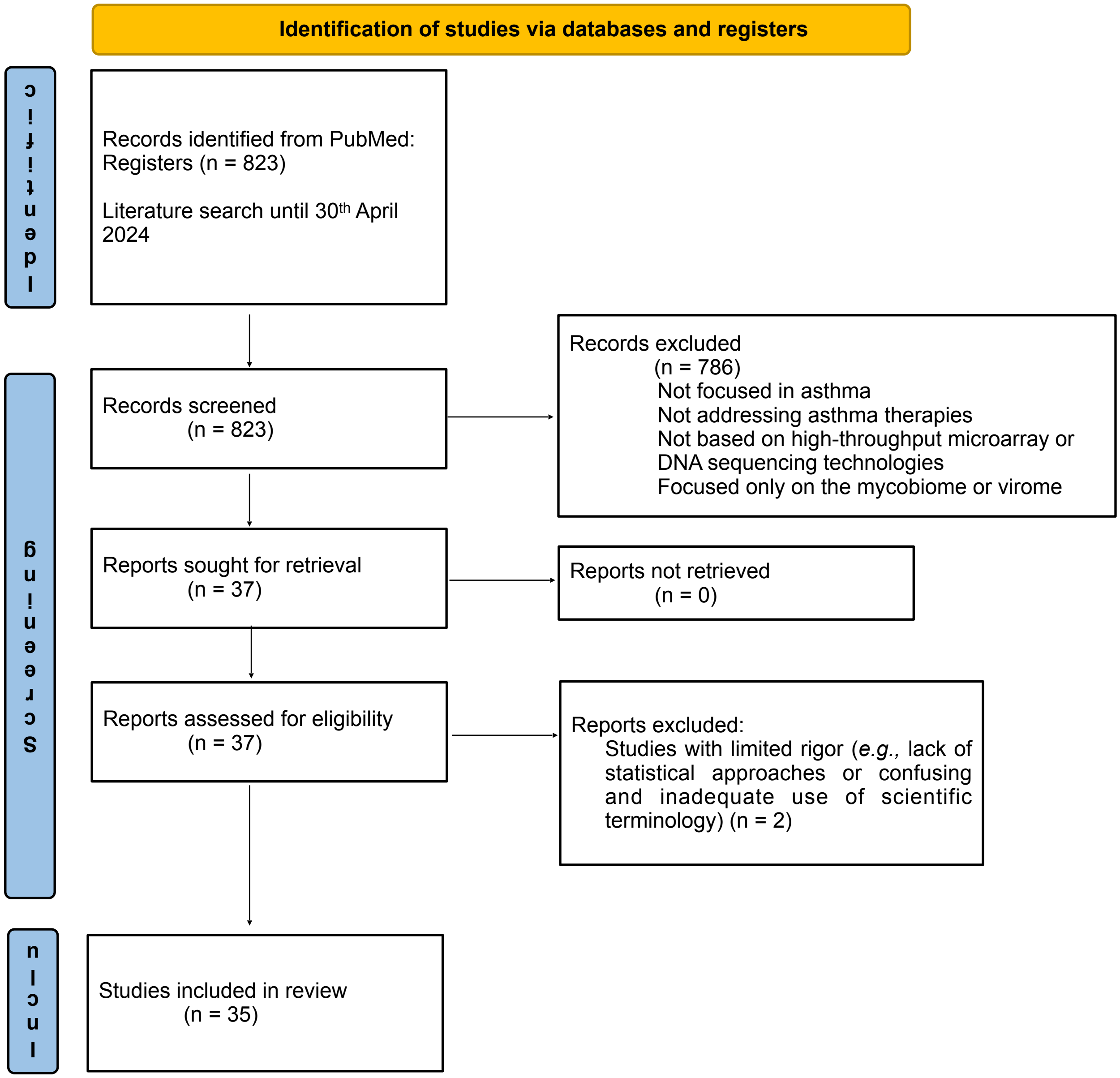

Supplementary Figure E1. Flowchart diagram for study selection based on the PRISMA 2020 guidelines.
